# Supplementary figures and images for: Evaluating socioeconomic inequalities in influenza vaccine uptake during the COVID-19 pandemic: A cohort study in Greater Manchester, England
Source: PLoS Med. 2023 Sep 26;20(9):e1004289. doi: 10.1371/journal.pmed.1004289 (PMC10522043; doi:10.1371/journal.pmed.1004289)

**S1 Fig. Study population flow chart****.**

**
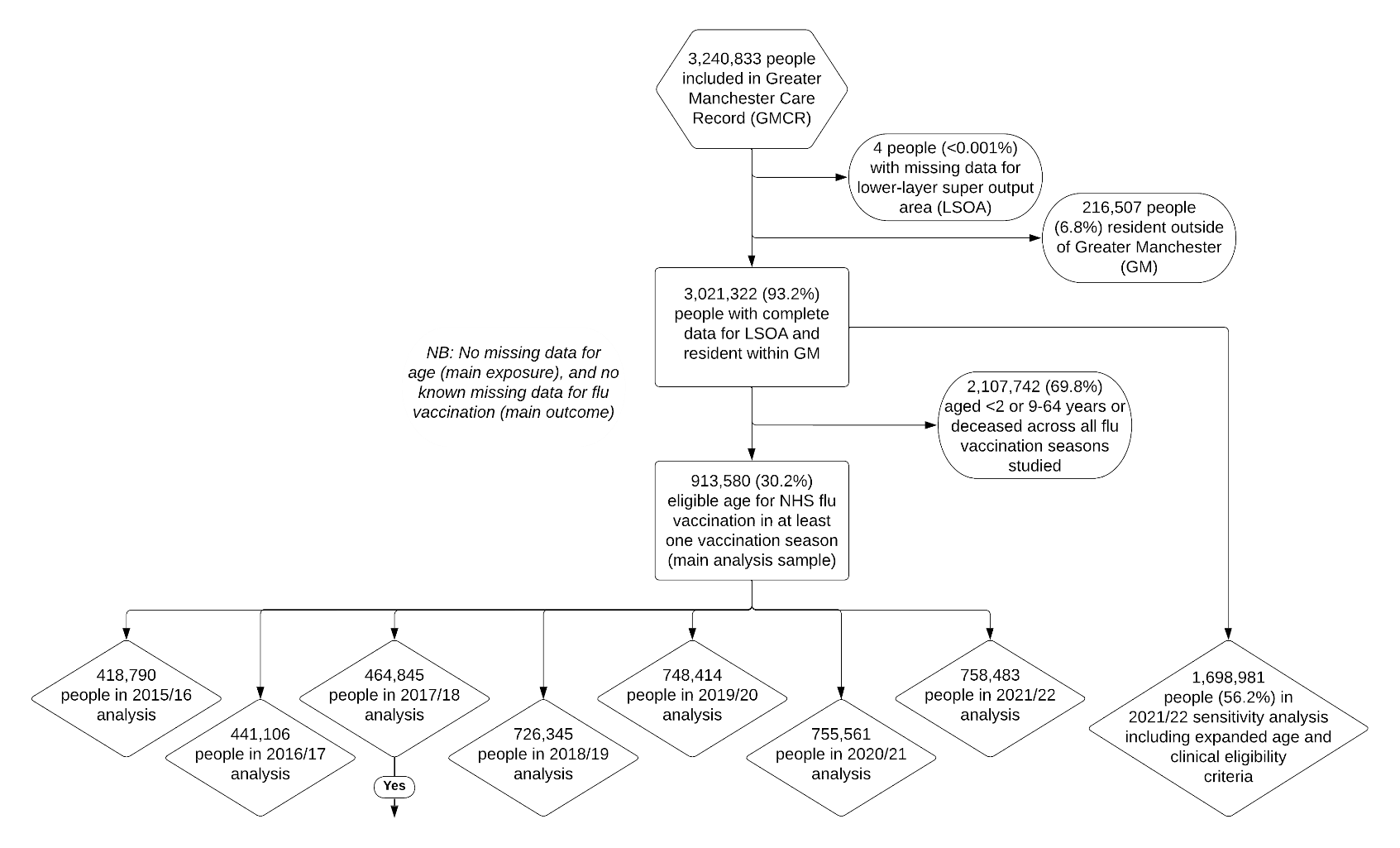
**


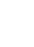


NHS: National Health Service

Supplement: S1 Fig — (DOCX) [file pmed.1004289.s001.docx]
