# Supplementary material for: Evaluating socioeconomic inequalities in influenza vaccine uptake during the COVID-19 pandemic: A cohort study in Greater Manchester, England
Source: PLoS Med. 2023 Sep 26;20(9):e1004289. doi: 10.1371/journal.pmed.1004289 (PMC10522043; doi:10.1371/journal.pmed.1004289)

**S2 Figure.** **Log(−log[survival]) versus log(time) plots by deprivation decile for Cox proportional hazards models by age group and vaccination season.***
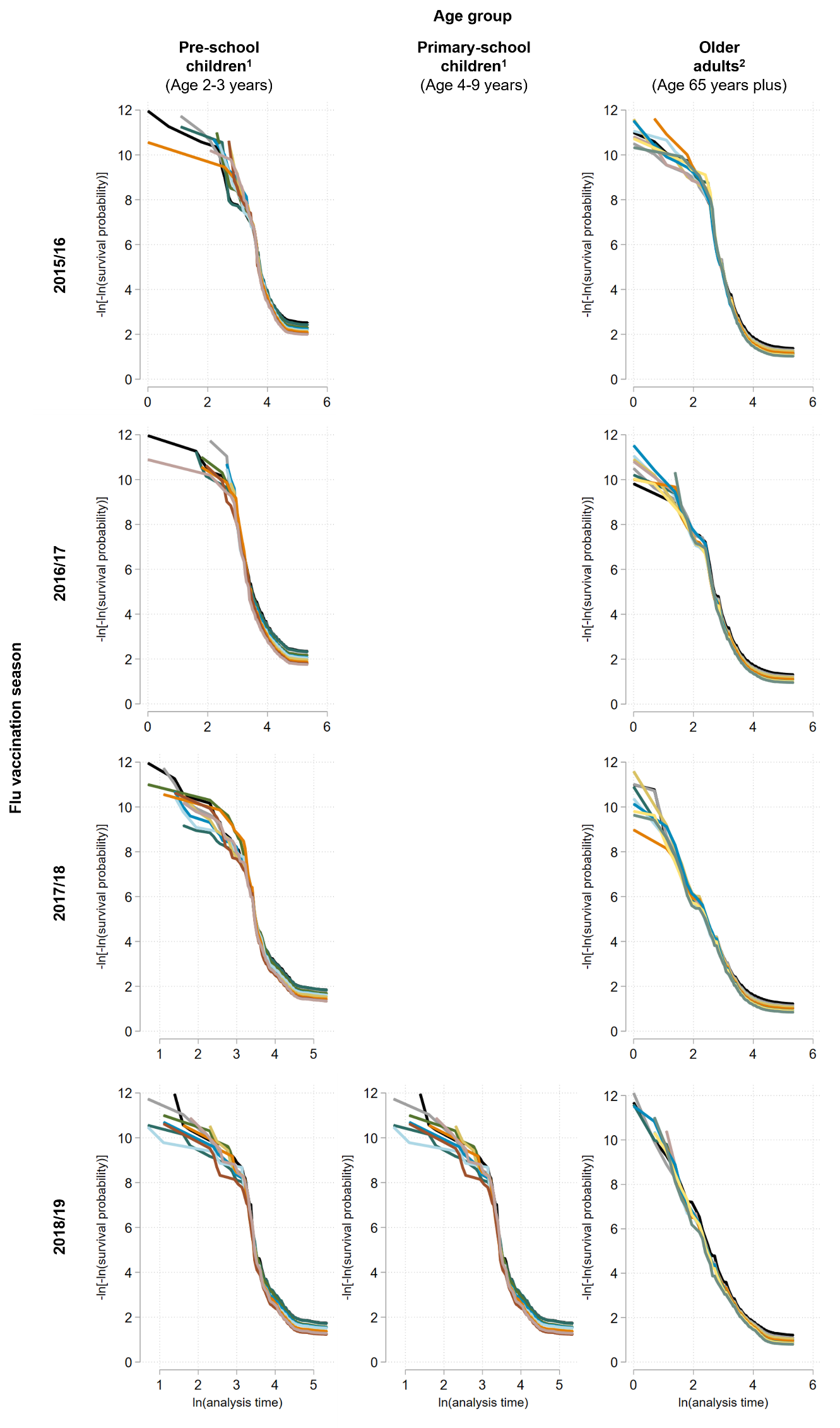
(continued over page)*


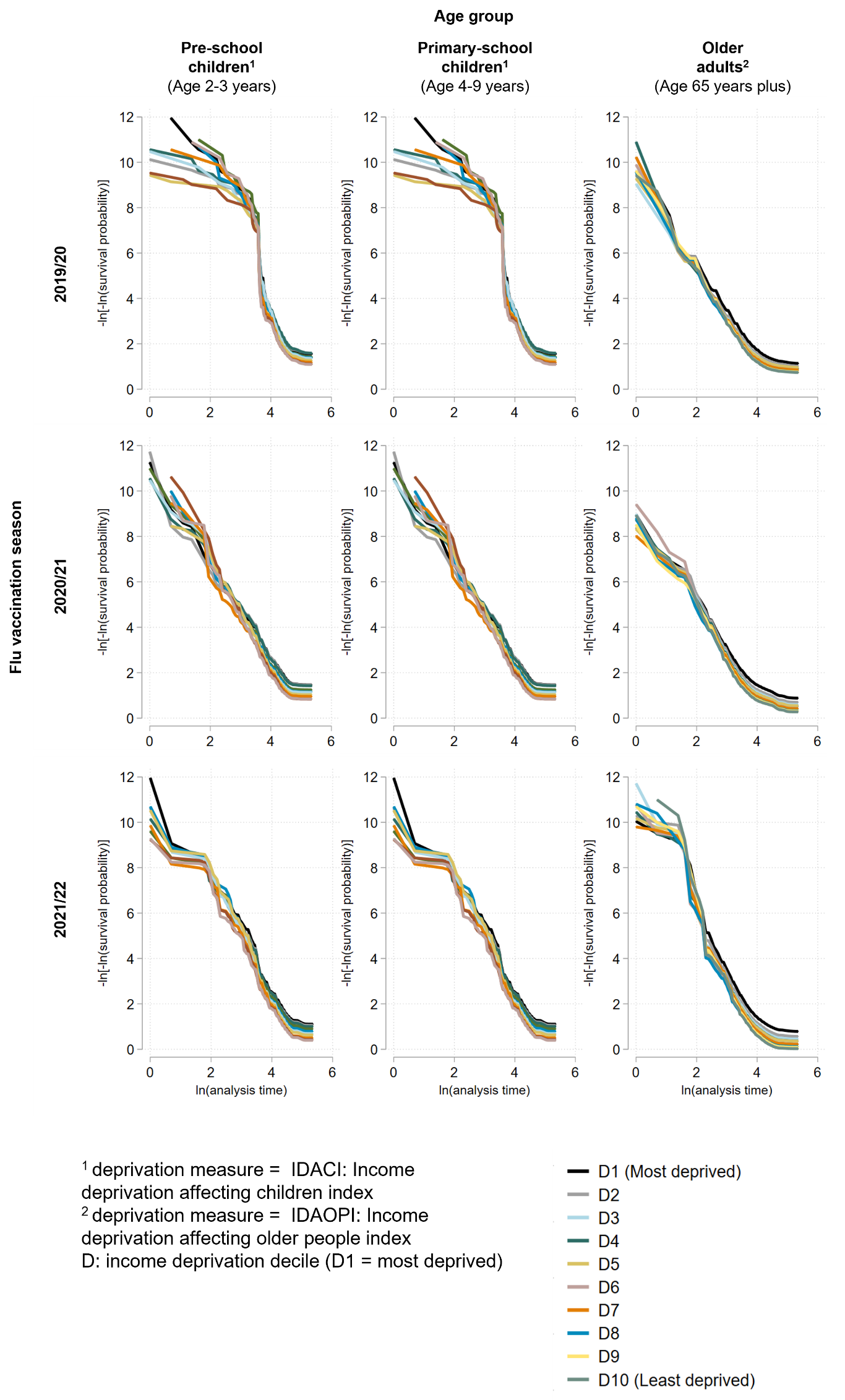

Supplement: S2 Fig — (DOCX) [file pmed.1004289.s002.docx]
