# Supplementary material for: Evaluating socioeconomic inequalities in influenza vaccine uptake during the COVID-19 pandemic: A cohort study in Greater Manchester, England
Source: PLoS Med. 2023 Sep 26;20(9):e1004289. doi: 10.1371/journal.pmed.1004289 (PMC10522043; doi:10.1371/journal.pmed.1004289)
Supplement: S2 Table — Results from Cox proportional hazards models are reported as hazard ratios with 95% confidence intervals. The reference group is D10 (least deprived areas) for each season. The vertical line indicates the onset of the pandemic. (DOCX) [file pmed.1004289.s005.docx]

**S2 Table. Relative** **unadjusted income deprivation-related inequalities in flu vaccine uptake amongst pre-school children (age 2-3 years) (for comparison with S1 Table).** Results from Cox proportional hazards models are reported as hazard ratios with 95% confidence intervals. The reference group is D10 (least deprived areas) for each season. The vertical line indicates the onset of the pandemic.

|  | **Flu vaccination season** | | | | | | |
| --- | --- | --- | --- | --- | --- | --- | --- |
|  | 2015/16 | 2016/17 | 2017/18 | 2018/19 | 2019/20 | 2020/21 | 2021/22 |
| **IDACI* decile** |  |  |  |  |  |  |  |
| D1 (Most deprived) | 0.54 | 0.53 | 0.54 | 0.52 | 0.55 | 0.48 | 0.50 |
|  | [0.51,0.56] | [0.50,0.55] | [0.51,0.56] | [0.50,0.55] | [0.52,0.57] | [0.46,0.50] | [0.48,0.52] |
| D2 | 0.58 | 0.53 | 0.54 | 0.53 | 0.54 | 0.49 | 0.50 |
|  | [0.55,0.62] | [0.51,0.56] | [0.51,0.56] | [0.51,0.56] | [0.51,0.56] | [0.47,0.51] | [0.48,0.53] |
| D3 | 0.56 | 0.53 | 0.53 | 0.51 | 0.56 | 0.51 | 0.51 |
|  | [0.53,0.60] | [0.50,0.56] | [0.50,0.56] | [0.48,0.54] | [0.53,0.59] | [0.49,0.54] | [0.49,0.54] |
| D4 | 0.66 | 0.66 | 0.63 | 0.63 | 0.62 | 0.61 | 0.60 |
|  | [0.62,0.70] | [0.62,0.70] | [0.60,0.67] | [0.59,0.66] | [0.59,0.66] | [0.58,0.64] | [0.57,0.64] |
| D5 | 0.69 | 0.71 | 0.66 | 0.65 | 0.69 | 0.66 | 0.62 |
|  | [0.65,0.74] | [0.67,0.76] | [0.62,0.70] | [0.61,0.69] | [0.65,0.74] | [0.63,0.70] | [0.59,0.66] |
| D6 | 0.73 | 0.68 | 0.70 | 0.70 | 0.74 | 0.71 | 0.66 |
|  | [0.68,0.78] | [0.63,0.72] | [0.65,0.74] | [0.66,0.75] | [0.70,0.79] | [0.67,0.75] | [0.62,0.70] |
| D7 | 0.81 | 0.81 | 0.80 | 0.82 | 0.77 | 0.73 | 0.71 |
|  | [0.76,0.86] | [0.76,0.86] | [0.76,0.85] | [0.77,0.87] | [0.72,0.82] | [0.69,0.78] | [0.67,0.75] |
| D8 | 0.80 | 0.84 | 0.88 | 0.87 | 0.89 | 0.87 | 0.83 |
|  | [0.75,0.85] | [0.79,0.90] | [0.83,0.93] | [0.82,0.93] | [0.84,0.94] | [0.83,0.92] | [0.78,0.89] |
| D9 | 0.96 | 0.95 | 0.97 | 1.00 | 0.99 | 0.92 | 0.95 |
|  | [0.90,1.02] | [0.90,1.01] | [0.91,1.02] | [0.95,1.06] | [0.94,1.05] | [0.87,0.97] | [0.89,1.00] |
| D10 (Least deprived) | Ref | Ref | Ref | Ref | Ref | Ref | Ref |
|  | - | - | - | - | - | - | - |
| **Observations** | 79278 | 78229 | 77750 | 77752 | 76267 | 73156 | 70419 |

Exponentiated coefficients (hazard ratios); 95% confidence intervals in brackets

* IDACI: Income deprivation affecting children index

D1 – D10: Deprivation deciles 1 - 10
