# Supplementary material for: Evaluating socioeconomic inequalities in influenza vaccine uptake during the COVID-19 pandemic: A cohort study in Greater Manchester, England
Source: PLoS Med. 2023 Sep 26;20(9):e1004289. doi: 10.1371/journal.pmed.1004289 (PMC10522043; doi:10.1371/journal.pmed.1004289)
Supplement: S4 Table — Results from Cox proportional hazards models are reported as hazard ratios with 95% confidence intervals. The reference group is D10 (least deprived areas). The vertical line indicates the onset of the pandemic. (DOCX) [file pmed.1004289.s007.docx]

**S4 Table. Relative unadjusted income deprivation-related inequalities in flu vaccine uptake amongst primary school children (age 4-9 years) (for comparison with S3 Table).** Results from Cox proportional hazards models are reported as hazard ratios with 95% confidence intervals. The reference groups are D10 (least deprived areas). The vertical line indicates the onset of the pandemic.

|  | **Flu vaccination season** | | | | |
| --- | --- | --- | --- | --- | --- |
|  | 2018/19 | | 2019/20 | 2020/21 | 2021/22 |
| **IDACI* decile** | |  | |  |  |
| D1 (Most deprived) | 0.61 | | 0.59 | 0.47 | 0.48 |
|  | [0.60,0.63] | | [0.58,0.61] | [0.46,0.48] | [0.47,0.49] |
| D2 | 0.62 | | 0.56 | 0.47 | 0.51 |
|  | [0.60,0.64] | | [0.55,0.58] | [0.46,0.48] | [0.50,0.52] |
| D3 | 0.63 | | 0.56 | 0.48 | 0.54 |
|  | [0.62,0.65] | | [0.54,0.57] | [0.47,0.50] | [0.53,0.55] |
| D4 | 0.76 | | 0.70 | 0.61 | 0.61 |
|  | [0.74,0.78] | | [0.68,0.73] | [0.59,0.63] | [0.59,0.62] |
| D5 | 0.79 | | 0.72 | 0.69 | 0.68 |
|  | [0.77,0.82] | | [0.70,0.75] | [0.66,0.71] | [0.66,0.70] |
| D6 | 0.80 | | 0.73 | 0.71 | 0.76 |
|  | [0.78,0.83] | | [0.71,0.76] | [0.68,0.73] | [0.73,0.78] |
| D7 | 0.95 | | 0.82 | 0.81 | 0.81 |
|  | [0.92,0.98] | | [0.80,0.85] | [0.78,0.83] | [0.79,0.83] |
| D8 | 0.94 | | 0.90 | 0.85 | 0.91 |
|  | [0.91,0.97] | | [0.88,0.93] | [0.83,0.88] | [0.89,0.94] |
| D9 | 1.10 | | 0.96 | 0.99 | 0.96 |
|  | [1.06,1.13] | | [0.93,0.99] | [0.97,1.02] | [0.93,0.98] |
| D10 (Least deprived) | Ref | | Ref | Ref | Ref |
|  | - | | - | - | - |
| **Observations** | 237386 | | 236651 | 235723 | 233277 |

Exponentiated coefficients (hazard ratios); 95% confidence intervals in brackets

* IDACI: Income deprivation affecting children index

D1 – D10: Deprivation deciles 1 - 10
