# Supplementary material for: Evaluating socioeconomic inequalities in influenza vaccine uptake during the COVID-19 pandemic: A cohort study in Greater Manchester, England
Source: PLoS Med. 2023 Sep 26;20(9):e1004289. doi: 10.1371/journal.pmed.1004289 (PMC10522043; doi:10.1371/journal.pmed.1004289)
Supplement: S5 Table — Results from Cox proportional hazards models adjusted by age are reported as hazard ratios with 95% confidence intervals. The reference groups are D10 (least deprived areas) and age 65–69 years for each season. The vertical line indicates the onset of the pandemic. Results also shown in Fig 2 in the main text. (DOCX) [file pmed.1004289.s008.docx]

**S5 Table. Relative** **age-adjusted income deprivation-related inequalities in flu vaccine uptake amongst older adults (age 65 years plus).** Results from Cox proportional hazards models adjusted by age are reported as hazard ratios with 95% confidence intervals. The reference groups are D10 (least deprived areas) and age 65-69 years for each season. The vertical line indicates the onset of the pandemic. Results also shown in Figure 2 in the main text.

|  | **Flu vaccination season** | | | | | | |
| --- | --- | --- | --- | --- | --- | --- | --- |
|  | 2015/16 | 2016/17 | 2017/18 | 2018/19 | 2019/20 | 2020/21 | 2021/22 |
| **IDAOPI* decile** |  |  |  |  |  |  |  |
| D1 (Most deprived) | 0.81 | 0.82 | 0.80 | 0.77 | 0.78 | 0.69 | 0.63 |
|  | [0.80,0.83] | [0.81,0.84] | [0.78,0.81] | [0.76,0.79] | [0.77,0.80] | [0.68,0.71] | [0.62,0.64] |
| D2 | 0.81 | 0.81 | 0.79 | 0.78 | 0.82 | 0.75 | 0.71 |
|  | [0.80,0.83] | [0.79,0.83] | [0.77,0.80] | [0.77,0.80] | [0.81,0.84] | [0.74,0.77] | [0.70,0.72] |
| D3 | 0.82 | 0.83 | 0.81 | 0.81 | 0.85 | 0.80 | 0.76 |
|  | [0.80,0.84] | [0.81,0.85] | [0.80,0.83] | [0.79,0.82] | [0.83,0.86] | [0.79,0.82] | [0.75,0.78] |
| D4 | 0.86 | 0.88 | 0.85 | 0.85 | 0.88 | 0.83 | 0.81 |
|  | [0.85,0.88] | [0.86,0.89] | [0.84,0.87] | [0.84,0.87] | [0.86,0.90] | [0.82,0.85] | [0.80,0.82] |
| D5 | 0.84 | 0.84 | 0.82 | 0.81 | 0.85 | 0.84 | 0.82 |
|  | [0.82,0.85] | [0.82,0.86] | [0.81,0.84] | [0.80,0.83] | [0.84,0.87] | [0.82,0.85] | [0.80,0.83] |
| D6 | 0.92 | 0.91 | 0.89 | 0.90 | 0.94 | 0.92 | 0.89 |
|  | [0.90,0.94] | [0.90,0.93] | [0.87,0.91] | [0.88,0.92] | [0.92,0.96] | [0.90,0.93] | [0.88,0.91] |
| D7 | 0.90 | 0.90 | 0.89 | 0.88 | 0.93 | 0.90 | 0.87 |
|  | [0.88,0.92] | [0.88,0.91] | [0.87,0.90] | [0.86,0.90] | [0.92,0.95] | [0.89,0.92] | [0.86,0.89] |
| D8 | 0.96 | 0.96 | 0.93 | 0.94 | 0.99 | 0.98 | 0.98 |
|  | [0.94,0.99] | [0.94,0.98] | [0.91,0.95] | [0.92,0.96] | [0.98,1.01] | [0.97,1.00] | [0.96,0.99] |
| D9 | 0.96 | 0.96 | 0.94 | 0.95 | 0.99 | 0.98 | 0.96 |
|  | [0.94,0.98] | [0.94,0.98] | [0.93,0.96] | [0.93,0.96] | [0.97,1.01] | [0.97,1.00] | [0.94,0.98] |
| D10 (Least deprived) | Ref | Ref | Ref | Ref | Ref | Ref | Ref |
|  | - | - | - | - | - | - | - |
| **Age group (years)** |  |  |  |  |  |  |  |
| 65-69 | Ref | Ref | Ref | Ref | Ref | Ref | Ref |
|  | - | - | - | - | - | - | - |
| 70-74 | 1.40 | 1.40 | 1.37 | 1.36 | 1.41 | 1.31 | 1.26 |
|  | [1.39,1.42] | [1.38,1.41] | [1.35,1.38] | [1.35,1.37] | [1.39,1.42] | [1.30,1.32] | [1.25,1.27] |
| 75-79 | 1.56 | 1.56 | 1.53 | 1.55 | 1.58 | 1.44 | 1.41 |
|  | [1.54,1.58] | [1.54,1.58] | [1.51,1.55] | [1.53,1.57] | [1.56,1.60] | [1.43,1.46] | [1.39,1.42] |
| 80+ | 1.37 | 1.40 | 1.40 | 1.44 | 1.47 | 1.33 | 1.36 |
|  | [1.36,1.39] | [1.39,1.42] | [1.38,1.42] | [1.42,1.45] | [1.45,1.49] | [1.32,1.35] | [1.35,1.37] |
|  |  |  |  |  |  |  |  |
| **Observations** | 339512 | 362877 | 387095 | 411207 | 435496 | 446682 | 454787 |

Exponentiated coefficients (hazard ratios); 95% confidence intervals in brackets

* IDAOPI: Income deprivation affecting older people index

D1 – D10: Deprivation deciles 1 - 10
