# Supplementary material for: Evaluating socioeconomic inequalities in influenza vaccine uptake during the COVID-19 pandemic: A cohort study in Greater Manchester, England
Source: PLoS Med. 2023 Sep 26;20(9):e1004289. doi: 10.1371/journal.pmed.1004289 (PMC10522043; doi:10.1371/journal.pmed.1004289)
Supplement: S6 Table — Results from Cox proportional hazards models are reported as hazard ratios with 95% confidence intervals. The reference group is D10 (least deprived areas) for each season. The vertical line indicates the onset of the pandemic. (DOCX) [file pmed.1004289.s009.docx]

**S6 Table. Relative** **unadjusted income deprivation-related inequalities in flu vaccine uptake amongst older adults (age 65 years plus) (for comparison with S5 Table).** Results from Cox proportional hazards models are reported as hazard ratios with 95% confidence intervals. The reference group is D10 (least deprived areas) for each season. The vertical line indicates the onset of the pandemic.

|  | **Flu vaccination season** | | | | | | |
| --- | --- | --- | --- | --- | --- | --- | --- |
|  | 2015/16 | 2016/17 | 2017/18 | 2018/19 | 2019/20 | 2020/21 | 2021/22 |
| **IDAOPI* decile** |  |  |  |  |  |  |  |
| D1 (Most deprived) | 0.81 | 0.82 | 0.79 | 0.76 | 0.77 | 0.68 | 0.61 |
|  | [0.80,0.83] | [0.80,0.83] | [0.77,0.80] | [0.75,0.78] | [0.75,0.78] | [0.67,0.69] | [0.60,0.62] |
| D2 | 0.81 | 0.81 | 0.78 | 0.78 | 0.81 | 0.75 | 0.70 |
|  | [0.80,0.83] | [0.79,0.82] | [0.77,0.80] | [0.76,0.79] | [0.80,0.83] | [0.73,0.76] | [0.69,0.71] |
| D3 | 0.82 | 0.83 | 0.81 | 0.81 | 0.84 | 0.80 | 0.75 |
|  | [0.81,0.84] | [0.81,0.85] | [0.80,0.83] | [0.79,0.82] | [0.83,0.86] | [0.78,0.81] | [0.74,0.77] |
| D4 | 0.87 | 0.88 | 0.85 | 0.85 | 0.87 | 0.83 | 0.80 |
|  | [0.85,0.88] | [0.86,0.89] | [0.83,0.87] | [0.83,0.87] | [0.86,0.89] | [0.81,0.84] | [0.79,0.82] |
| D5 | 0.84 | 0.84 | 0.82 | 0.81 | 0.85 | 0.83 | 0.81 |
|  | [0.82,0.86] | [0.82,0.86] | [0.81,0.84] | [0.79,0.83] | [0.83,0.86] | [0.82,0.85] | [0.80,0.82] |
| D6 | 0.92 | 0.91 | 0.89 | 0.90 | 0.94 | 0.91 | 0.89 |
|  | [0.90,0.94] | [0.89,0.93] | [0.87,0.90] | [0.88,0.92] | [0.92,0.96] | [0.90,0.93] | [0.87,0.90] |
| D7 | 0.90 | 0.89 | 0.88 | 0.88 | 0.93 | 0.90 | 0.87 |
|  | [0.88,0.92] | [0.88,0.91] | [0.87,0.90] | [0.86,0.89] | [0.91,0.95] | [0.88,0.91] | [0.86,0.89] |
| D8 | 0.96 | 0.96 | 0.93 | 0.94 | 0.99 | 0.98 | 0.97 |
|  | [0.94,0.98] | [0.94,0.98] | [0.91,0.95] | [0.92,0.95] | [0.97,1.01] | [0.96,1.00] | [0.96,0.99] |
| D9 | 0.96 | 0.96 | 0.95 | 0.95 | 0.99 | 0.98 | 0.96 |
|  | [0.94,0.98] | [0.94,0.98] | [0.93,0.96] | [0.93,0.97] | [0.97,1.01] | [0.97,1.00] | [0.94,0.97] |
| D10 (Least deprived) | Ref | Ref | Ref | Ref | Ref | Ref | Ref |
|  | - | - | - | - | - | - | - |
| **Observations** | 339512 | 362877 | 387095 | 411207 | 435496 | 446682 | 454787 |

Exponentiated coefficients (hazard ratios); 95% confidence intervals in brackets

* IDAOPI: Income deprivation affecting older people index

D1 – D10: Deprivation deciles 1 - 10
