# Supplementary material for: Evaluating socioeconomic inequalities in influenza vaccine uptake during the COVID-19 pandemic: A cohort study in Greater Manchester, England
Source: PLoS Med. 2023 Sep 26;20(9):e1004289. doi: 10.1371/journal.pmed.1004289 (PMC10522043; doi:10.1371/journal.pmed.1004289)
Supplement: S9 Table — Results from Cox proportional hazards models adjusted by age are reported as hazard ratios with 95% confidence intervals. The reference groups are D10 (least deprived areas) and age 65–69 years for each season. The vertical line indicates the onset of the pandemic. (DOCX) [file pmed.1004289.s012.docx]

**S9 Table. Relative** **age-adjusted income deprivation-related inequalities in flu vaccine uptake amongst older adults (age 65 years plus) – sensitivity analysis excluding individuals who died during the study period.** Results from Cox proportional hazards models adjusted by age are reported as hazard ratios with 95% confidence intervals. The reference groups are D10 (least deprived areas) and age 65-69 years for each season. The vertical line indicates the onset of the pandemic.

|  | **Flu vaccination season** | | | | | | |
| --- | --- | --- | --- | --- | --- | --- | --- |
|  | 2015/16 | 2016/17 | 2017/18 | 2018/19 | 2019/20 | 2020/21 | 2021/22 |
| **IDAOPI* decile** |  |  |  |  |  |  |  |
| D1 (Most deprived) | 0.83 | 0.83 | 0.81 | 0.79 | 0.79 | 0.69 | 0.63 |
|  | [0.81,0.85] | [0.82,0.85] | [0.79,0.82] | [0.77,0.80] | [0.78,0.81] | [0.68,0.71] | [0.62,0.64] |
| D2 | 0.84 | 0.83 | 0.81 | 0.80 | 0.84 | 0.76 | 0.71 |
|  | [0.82,0.86] | [0.82,0.85] | [0.79,0.83] | [0.79,0.82] | [0.82,0.85] | [0.75,0.77] | [0.70,0.73] |
| D3 | 0.85 | 0.86 | 0.84 | 0.83 | 0.87 | 0.81 | 0.77 |
|  | [0.83,0.87] | [0.84,0.88] | [0.82,0.85] | [0.82,0.85] | [0.85,0.89] | [0.80,0.83] | [0.75,0.78] |
| D4 | 0.89 | 0.90 | 0.88 | 0.88 | 0.90 | 0.84 | 0.81 |
|  | [0.87,0.91] | [0.88,0.92] | [0.86,0.90] | [0.86,0.89] | [0.89,0.92] | [0.83,0.86] | [0.80,0.83] |
| D5 | 0.86 | 0.86 | 0.84 | 0.83 | 0.87 | 0.84 | 0.82 |
|  | [0.84,0.88] | [0.84,0.88] | [0.82,0.86] | [0.81,0.84] | [0.85,0.89] | [0.83,0.86] | [0.81,0.83] |
| D6 | 0.92 | 0.92 | 0.89 | 0.90 | 0.95 | 0.92 | 0.89 |
|  | [0.90,0.94] | [0.90,0.94] | [0.87,0.91] | [0.89,0.92] | [0.93,0.97] | [0.90,0.94] | [0.88,0.91] |
| D7 | 0.91 | 0.90 | 0.89 | 0.89 | 0.94 | 0.91 | 0.88 |
|  | [0.89,0.93] | [0.89,0.92] | [0.87,0.91] | [0.87,0.90] | [0.93,0.96] | [0.89,0.92] | [0.86,0.89] |
| D8 | 0.97 | 0.97 | 0.94 | 0.94 | 1.00 | 0.98 | 0.98 |
|  | [0.95,1.00] | [0.95,0.99] | [0.92,0.96] | [0.93,0.96] | [0.98,1.02] | [0.97,1.00] | [0.96,0.99] |
| D9 | 0.97 | 0.98 | 0.95 | 0.96 | 1.00 | 0.99 | 0.96 |
|  | [0.95,0.99] | [0.96,1.00] | [0.93,0.97] | [0.94,0.98] | [0.98,1.02] | [0.97,1.01] | [0.94,0.98] |
| D10 (Least deprived) | Ref | Ref | Ref | Ref | Ref | Ref | Ref |
|  | - | - | - | - | - | - | - |
| **Age group (years)** |  |  |  |  |  |  |  |
| 65-69 | Ref | Ref | Ref | Ref | Ref | Ref | Ref |
|  | - | - | - | - | - | - | - |
| 70-74 | 1.44 | 1.43 | 1.40 | 1.39 | 1.44 | 1.32 | 1.26 |
|  | [1.43,1.46] | [1.42,1.45] | [1.38,1.42] | [1.37,1.40] | [1.42,1.45] | [1.31,1.33] | [1.25,1.27] |
| 75-79 | 1.67 | 1.66 | 1.62 | 1.63 | 1.65 | 1.47 | 1.42 |
|  | [1.65,1.69] | [1.64,1.68] | [1.60,1.64] | [1.61,1.65] | [1.63,1.67] | [1.45,1.49] | [1.40,1.43] |
| 80+ | 1.63 | 1.66 | 1.64 | 1.67 | 1.69 | 1.41 | 1.39 |
|  | [1.61,1.65] | [1.63,1.68] | [1.62,1.65] | [1.65,1.69] | [1.67,1.71] | [1.40,1.42] | [1.37,1.40] |
|  |  |  |  |  |  |  |  |
| **Observations** | 294316 | 316728 | 340066 | 363314 | 386876 | 411978 | 440778 |

Exponentiated coefficients (hazard ratios); 95% confidence intervals in brackets

* IDAOPI: Income deprivation affecting older people index

D1 – D10: Deprivation deciles 1 - 10
