# Supplementary material for: Evaluating socioeconomic inequalities in influenza vaccine uptake during the COVID-19 pandemic: A cohort study in Greater Manchester, England
Source: PLoS Med. 2023 Sep 26;20(9):e1004289. doi: 10.1371/journal.pmed.1004289 (PMC10522043; doi:10.1371/journal.pmed.1004289)
Supplement: S10 Table — Results from Cox proportional hazards models adjusted by age are reported as hazard ratios with 95% confidence intervals. The reference groups are D10 (least deprived areas) and age 2 years for each season. The vertical line indicates the onset of the pandemic. (DOCX) [file pmed.1004289.s013.docx]

**S10 Table. Relative** **age-adjusted multiple deprivation-related inequalities in flu vaccine uptake amongst pre-school children (age 2-3 years) – sensitivity analysis using the index of multiple deprivation (IMD) as an alternative measure of deprivation.** Results from Cox proportional hazards models adjusted by age are reported as hazard ratios with 95% confidence intervals. The reference groups are D10 (least deprived areas) and age 2 years for each season. The vertical line indicates the onset of the pandemic.

|  | **Flu vaccination season** | | | | | | |
| --- | --- | --- | --- | --- | --- | --- | --- |
|  | 2015/16 | 2016/17 | 2017/18 | 2018/19 | 2019/20 | 2020/21 | 2021/22 |
| **Deprivation** |  |  |  |  |  |  |  |
| D1 (Most deprived) | 0.53 | 0.46 | 0.44 | 0.47 | 0.48 | 0.44 | 0.45 |
|  | [0.50,0.57] | [0.44,0.49] | [0.42,0.47] | [0.44,0.49] | [0.45,0.50] | [0.41,0.46] | [0.43,0.48] |
| D2 | 0.55 | 0.46 | 0.44 | 0.46 | 0.47 | 0.42 | 0.43 |
|  | [0.52,0.59] | [0.43,0.49] | [0.42,0.47] | [0.43,0.48] | [0.44,0.50] | [0.40,0.45] | [0.40,0.45] |
| D3 | 0.60 | 0.54 | 0.52 | 0.55 | 0.55 | 0.52 | 0.52 |
|  | [0.57,0.65] | [0.51,0.58] | [0.49,0.55] | [0.52,0.59] | [0.51,0.58] | [0.49,0.56] | [0.48,0.55] |
| D4 | 0.66 | 0.59 | 0.56 | 0.59 | 0.59 | 0.55 | 0.55 |
|  | [0.61,0.71] | [0.55,0.63] | [0.53,0.60] | [0.55,0.63] | [0.55,0.63] | [0.51,0.58] | [0.52,0.59] |
| D5 | 0.75 | 0.66 | 0.60 | 0.66 | 0.66 | 0.67 | 0.63 |
|  | [0.70,0.81] | [0.61,0.71] | [0.56,0.65] | [0.62,0.71] | [0.61,0.70] | [0.63,0.71] | [0.58,0.67] |
| D6 | 0.80 | 0.69 | 0.70 | 0.70 | 0.71 | 0.65 | 0.64 |
|  | [0.74,0.86] | [0.64,0.74] | [0.65,0.74] | [0.65,0.75] | [0.66,0.76] | [0.60,0.69] | [0.59,0.69] |
| D7 | 0.82 | 0.73 | 0.73 | 0.80 | 0.79 | 0.78 | 0.75 |
|  | [0.76,0.88] | [0.68,0.79] | [0.68,0.78] | [0.75,0.86] | [0.74,0.84] | [0.73,0.83] | [0.69,0.80] |
| D8 | 0.86 | 0.81 | 0.79 | 0.86 | 0.82 | 0.80 | 0.80 |
|  | [0.80,0.92] | [0.75,0.86] | [0.74,0.85] | [0.81,0.92] | [0.77,0.87] | [0.75,0.85] | [0.75,0.86] |
| D9 | 1.01 | 0.88 | 0.85 | 0.94 | 0.89 | 0.89 | 0.88 |
|  | [0.94,1.08] | [0.82,0.94] | [0.80,0.91] | [0.89,1.01] | [0.83,0.95] | [0.84,0.95] | [0.82,0.94] |
| D10 (Least deprived) | Ref | Ref | Ref | Ref | Ref | Ref | Ref |
|  | - | - | - | - | - | - | - |
| **Age (years)** |  |  |  |  |  |  |  |
| 2 | Ref | Ref | Ref | Ref | Ref | Ref | Ref |
|  | - | - | - | - | - | - | - |
| 3 | 1.63 | 1.69 | 1.72 | 1.72 | 1.74 | 1.75 | 1.78 |
|  | [1.59,1.68] | [1.65,1.74] | [1.68,1.77] | [1.67,1.76] | [1.70,1.79] | [1.71,1.79] | [1.74,1.83] |
|  |  |  |  |  |  |  |  |
| **Observations** | 79278 | 78229 | 77750 | 77752 | 76267 | 73156 | 70419 |

Exponentiated coefficients (hazard ratios); 95% confidence intervals in brackets

D1 – D10: Deprivation deciles 1 - 10
