# Supplementary material for: Evaluating socioeconomic inequalities in influenza vaccine uptake during the COVID-19 pandemic: A cohort study in Greater Manchester, England
Source: PLoS Med. 2023 Sep 26;20(9):e1004289. doi: 10.1371/journal.pmed.1004289 (PMC10522043; doi:10.1371/journal.pmed.1004289)
Supplement: S11 Table — Results from Cox proportional hazards models adjusted by age are reported as hazard ratios with 95% confidence intervals. The reference groups are D10 (least deprived areas) and age 4 years for each season. The vertical line indicates the onset of the pandemic. (DOCX) [file pmed.1004289.s014.docx]

**S11 Table. Relative** **age-adjusted multiple deprivation-related inequalities in flu vaccine uptake amongst primary school children (age 4-9 years) – sensitivity analysis using the index of multiple deprivation (IMD) as an alternative measure of deprivation.** Results from Cox proportional hazards models adjusted by age are reported as hazard ratios with 95% confidence intervals. The reference groups are D10 (least deprived areas) and age 4 years for each season. The vertical line indicates the onset of the pandemic.

|  | **Flu vaccination season** | | | | | | |
| --- | --- | --- | --- | --- | --- | --- | --- |
|  | 2018/19 | | 2019/20 | 2020/21 | | 2021/22 | |
| **Deprivation** | |  | |  |  | |  |
| D1 (Most deprived) | 0.56 | | 0.55 | 0.42 | | 0.44 | |
|  | [0.54,0.57] | | [0.53,0.56] | [0.41,0.43] | | [0.43,0.45] | |
| D2 | 0.55 | | 0.52 | 0.42 | | 0.47 | |
|  | [0.53,0.57] | | [0.50,0.53] | [0.41,0.43] | | [0.46,0.49] | |
| D3 | 0.65 | | 0.60 | 0.53 | | 0.56 | |
|  | [0.63,0.67] | | [0.58,0.62] | [0.52,0.55] | | [0.55,0.58] | |
| D4 | 0.71 | | 0.65 | 0.59 | | 0.59 | |
|  | [0.68,0.74] | | [0.63,0.67] | [0.57,0.61] | | [0.57,0.61] | |
| D5 | 0.81 | | 0.73 | 0.70 | | 0.76 | |
|  | [0.78,0.84] | | [0.70,0.76] | [0.68,0.72] | | [0.73,0.78] | |
| D6 | 0.82 | | 0.77 | 0.70 | | 0.75 | |
|  | [0.79,0.85] | | [0.75,0.80] | [0.67,0.72] | | [0.72,0.77] | |
| D7 | 0.87 | | 0.86 | 0.83 | | 0.78 | |
|  | [0.84,0.90] | | [0.83,0.89] | [0.80,0.86] | | [0.76,0.81] | |
| D8 | 0.96 | | 0.90 | 0.88 | | 0.90 | |
|  | [0.93,1.00] | | [0.87,0.93] | [0.85,0.91] | | [0.88,0.93] | |
| D9 | 1.00 | | 0.94 | 0.97 | | 0.94 | |
|  | [0.96,1.03] | | [0.91,0.98] | [0.94,1.00] | | [0.91,0.97] | |
| D10 (Least deprived) | Ref | | Ref | Ref | | Ref | |
|  | - | | - | - | | - | |
| **Age (years)** |  | |  |  | |  | |
| 4 | Ref | | Ref | Ref | | Ref | |
|  | - | | - | - | | - | |
| 5 | 1.06 | | 1.10 | 0.92 | | 1.16 | |
|  | [1.04,1.09] | | [1.07,1.12] | [0.90,0.94] | | [1.14,1.18] | |
| 6 | 1.04 | | 1.07 | 0.92 | | 1.16 | |
|  | [1.02,1.07] | | [1.05,1.10] | [0.90,0.94] | | [1.14,1.19] | |
| 7 | 1.03 | | 1.04 | 0.92 | | 1.18 | |
|  | [1.00,1.05] | | [1.02,1.06] | [0.90,0.94] | | [1.16,1.21] | |
| 8 | 1.01 | | 1.02 | 0.91 | | 1.16 | |
|  | [0.98,1.03] | | [0.99,1.04] | [0.89,0.93] | | [1.14,1.18] | |
| 9 | 0.95 | | 0.97 | 0.90 | | 1.14 | |
|  | [0.93,0.98] | | [0.95,0.99] | [0.88,0.92] | | [1.12,1.16] | |
|  |  | |  |  | |  | |
| **Observations** | 237386 | | 236651 | 235723 | | 233277 | |

Exponentiated coefficients (hazard ratios); 95% confidence intervals in brackets

D1 – D10: Deprivation deciles 1 - 10
