# Supplementary material for: Evaluating socioeconomic inequalities in influenza vaccine uptake during the COVID-19 pandemic: A cohort study in Greater Manchester, England
Source: PLoS Med. 2023 Sep 26;20(9):e1004289. doi: 10.1371/journal.pmed.1004289 (PMC10522043; doi:10.1371/journal.pmed.1004289)
Supplement: S12 Table — Results from Cox proportional hazards models adjusted by age are reported as hazard ratios with 95% confidence intervals. The reference groups are D10 (least deprived areas) and age 65–69 years for each season. The vertical line indicates the onset of the pandemic. (DOCX) [file pmed.1004289.s015.docx]

**S12 Table. Relative** **age-adjusted multiple deprivation-related inequalities in flu vaccine uptake amongst older adults (age 65 years plus) – sensitivity analysis using the index of multiple deprivation (IMD) as an alternative measure of deprivation.** Results from Cox proportional hazards models adjusted by age are reported as hazard ratios with 95% confidence intervals. The reference groups are D10 (least deprived areas) and age 65-69 years for each season. The vertical line indicates the onset of the pandemic.

|  | **Flu vaccination season** | | | | | | |
| --- | --- | --- | --- | --- | --- | --- | --- |
|  | 2015/16 | 2016/17 | 2017/18 | 2018/19 | 2019/20 | 2020/21 | 2021/22 |
| **Deprivation** |  |  |  |  |  |  |  |
| D1 (Most deprived) | 0.83 | 0.83 | 0.81 | 0.81 | 0.81 | 0.74 | 0.68 |
|  | [0.81,0.85] | [0.81,0.84] | [0.80,0.83] | [0.80,0.83] | [0.80,0.83] | [0.73,0.75] | [0.67,0.69] |
| D2 | 0.79 | 0.79 | 0.78 | 0.79 | 0.80 | 0.75 | 0.71 |
|  | [0.78,0.81] | [0.77,0.80] | [0.76,0.79] | [0.78,0.81] | [0.79,0.82] | [0.74,0.77] | [0.70,0.72] |
| D3 | 0.85 | 0.85 | 0.84 | 0.84 | 0.85 | 0.82 | 0.78 |
|  | [0.84,0.87] | [0.83,0.87] | [0.83,0.86] | [0.83,0.86] | [0.83,0.86] | [0.81,0.83] | [0.77,0.80] |
| D4 | 0.86 | 0.84 | 0.84 | 0.86 | 0.87 | 0.82 | 0.79 |
|  | [0.84,0.88] | [0.83,0.86] | [0.82,0.86] | [0.84,0.88] | [0.85,0.88] | [0.80,0.83] | [0.77,0.80] |
| D5 | 0.87 | 0.86 | 0.84 | 0.85 | 0.87 | 0.86 | 0.85 |
|  | [0.85,0.89] | [0.84,0.88] | [0.83,0.86] | [0.84,0.87] | [0.86,0.89] | [0.84,0.87] | [0.83,0.86] |
| D6 | 0.90 | 0.89 | 0.89 | 0.92 | 0.92 | 0.91 | 0.89 |
|  | [0.88,0.92] | [0.87,0.91] | [0.88,0.91] | [0.91,0.94] | [0.90,0.93] | [0.90,0.93] | [0.87,0.90] |
| D7 | 0.92 | 0.92 | 0.91 | 0.92 | 0.94 | 0.92 | 0.92 |
|  | [0.90,0.94] | [0.90,0.94] | [0.89,0.93] | [0.90,0.94] | [0.92,0.95] | [0.90,0.93] | [0.90,0.93] |
| D8 | 0.92 | 0.91 | 0.90 | 0.90 | 0.94 | 0.93 | 0.91 |
|  | [0.90,0.94] | [0.90,0.93] | [0.88,0.91] | [0.89,0.92] | [0.92,0.95] | [0.92,0.95] | [0.90,0.93] |
| D9 | 0.94 | 0.92 | 0.92 | 0.95 | 0.95 | 0.97 | 0.97 |
|  | [0.92,0.96] | [0.90,0.93] | [0.90,0.93] | [0.93,0.96] | [0.93,0.97] | [0.95,0.98] | [0.95,0.98] |
| D10 (Least deprived) | Ref | Ref | Ref | Ref | Ref | Ref | Ref |
|  | - | - | - | - | - | - | - |
| **Age group (years)** |  |  |  |  |  |  |  |
| 65-69 | Ref | Ref | Ref | Ref | Ref | Ref | Ref |
|  | - | - | - | - | - | - | - |
| 70-74 | 1.40 | 1.40 | 1.37 | 1.36 | 1.41 | 1.31 | 1.26 |
|  | [1.39,1.42] | [1.38,1.41] | [1.36,1.38] | [1.35,1.38] | [1.40,1.42] | [1.30,1.33] | [1.25,1.27] |
| 75-79 | 1.56 | 1.56 | 1.53 | 1.55 | 1.58 | 1.45 | 1.41 |
|  | [1.54,1.58] | [1.54,1.58] | [1.51,1.55] | [1.54,1.57] | [1.56,1.60] | [1.43,1.46] | [1.40,1.43] |
| 80+ | 1.37 | 1.40 | 1.40 | 1.44 | 1.47 | 1.33 | 1.36 |
|  | [1.35,1.39] | [1.38,1.42] | [1.38,1.41] | [1.42,1.45] | [1.45,1.48] | [1.32,1.35] | [1.35,1.38] |
|  |  |  |  |  |  |  |  |
| **Observations** | 339512 | 362877 | 387095 | 411207 | 435496 | 446682 | 454787 |

Exponentiated coefficients (hazard ratios); 95% confidence intervals in brackets

D1 – D10: Deprivation deciles 1 - 10
