# Supplementary material for: Evaluating socioeconomic inequalities in influenza vaccine uptake during the COVID-19 pandemic: A cohort study in Greater Manchester, England
Source: PLoS Med. 2023 Sep 26;20(9):e1004289. doi: 10.1371/journal.pmed.1004289 (PMC10522043; doi:10.1371/journal.pmed.1004289)
Supplement: S13 Table — Results from Cox proportional hazards models adjusted by age are reported as hazard ratios with 95% confidence intervals. The reference groups are D10 (least deprived areas) and age 2 years for each season. The vertical line indicates the onset of the pandemic. (DOCX) [file pmed.1004289.s016.docx]

**S13 Table. Relative** **age-adjusted income deprivation-related inequalities in flu vaccine uptake amongst pre-school children (age 2-3 years) – sensitivity analysis excluding children on the border of age-based vaccine eligibility (i.e.: excluding age 3/4 years).** Results from Cox proportional hazards models adjusted by age are reported as hazard ratios with 95% confidence intervals. The reference groups are D10 (least deprived areas) and age 2 years for each season. The vertical line indicates the onset of the pandemic.

|  | **Flu vaccination season** | | | | | | |
| --- | --- | --- | --- | --- | --- | --- | --- |
|  | 2015/16 | 2016/17 | 2017/18 | 2018/19 | 2019/20 | 2020/21 | 2021/22 |
| **IDACI* decile** |  |  |  |  |  |  |  |
| D1 (Most deprived) | 0.52 | 0.49 | 0.52 | 0.53 | 0.53 | 0.48 | 0.54 |
|  | [0.48,0.56] | [0.45,0.53] | [0.48,0.56] | [0.49,0.57] | [0.49,0.57] | [0.45,0.52] | [0.50,0.58] |
| D2 | 0.58 | 0.49 | 0.55 | 0.54 | 0.52 | 0.50 | 0.52 |
|  | [0.54,0.63] | [0.46,0.53] | [0.51,0.60] | [0.50,0.58] | [0.48,0.56] | [0.46,0.53] | [0.48,0.57] |
| D3 | 0.56 | 0.51 | 0.53 | 0.53 | 0.55 | 0.53 | 0.53 |
|  | [0.51,0.61] | [0.47,0.56] | [0.49,0.58] | [0.48,0.57] | [0.50,0.60] | [0.49,0.58] | [0.49,0.58] |
| D4 | 0.66 | 0.61 | 0.65 | 0.64 | 0.60 | 0.62 | 0.63 |
|  | [0.60,0.73] | [0.56,0.67] | [0.60,0.71] | [0.58,0.70] | [0.55,0.65] | [0.57,0.67] | [0.57,0.69] |
| D5 | 0.68 | 0.68 | 0.66 | 0.67 | 0.71 | 0.63 | 0.64 |
|  | [0.61,0.75] | [0.62,0.75] | [0.60,0.72] | [0.60,0.73] | [0.64,0.78] | [0.58,0.69] | [0.58,0.71] |
| D6 | 0.69 | 0.65 | 0.75 | 0.72 | 0.74 | 0.70 | 0.72 |
|  | [0.61,0.77] | [0.58,0.72] | [0.68,0.82] | [0.65,0.79] | [0.67,0.82] | [0.63,0.77] | [0.64,0.80] |
| D7 | 0.83 | 0.77 | 0.81 | 0.84 | 0.79 | 0.72 | 0.73 |
|  | [0.75,0.92] | [0.70,0.85] | [0.74,0.89] | [0.77,0.93] | [0.72,0.87] | [0.66,0.79] | [0.66,0.81] |
| D8 | 0.79 | 0.85 | 0.88 | 0.88 | 0.88 | 0.86 | 0.86 |
|  | [0.72,0.88] | [0.78,0.94] | [0.80,0.97] | [0.80,0.97] | [0.80,0.97] | [0.79,0.94] | [0.78,0.95] |
| D9 | 0.91 | 0.95 | 0.96 | 1.02 | 0.98 | 0.94 | 0.95 |
|  | [0.82,1.00] | [0.87,1.04] | [0.88,1.05] | [0.93,1.11] | [0.89,1.07] | [0.86,1.02] | [0.86,1.04] |
| D10 (Least deprived) | Ref | Ref | Ref | Ref | Ref | Ref | Ref |
|  | - | - | - | - | - | - | - |
| **Age (years)** |  |  |  |  |  |  |  |
| 2 | Ref | Ref | Ref | Ref | Ref | Ref | Ref |
|  | - | - | - | - | - | - | - |
|  |  |  |  |  |  |  |  |
| **Observations** | 39244 | 38978 | 38775 | 38971 | 37298 | 35861 | 34565 |

Exponentiated coefficients (hazard ratios); 95% confidence intervals in brackets

* IDACI: Income deprivation affecting children index

D1 – D10: Deprivation deciles 1 - 10
