# Supplementary material for: Evaluating socioeconomic inequalities in influenza vaccine uptake during the COVID-19 pandemic: A cohort study in Greater Manchester, England
Source: PLoS Med. 2023 Sep 26;20(9):e1004289. doi: 10.1371/journal.pmed.1004289 (PMC10522043; doi:10.1371/journal.pmed.1004289)
Supplement: S14 Table — Results from Cox proportional hazards models adjusted by age are reported as hazard ratios with 95% confidence intervals. The reference groups are D10 (least deprived areas) and age 4 years for each season. The vertical line indicates the onset of the pandemic. (DOCX) [file pmed.1004289.s017.docx]

**S14 Table. Relative** **age-adjusted income deprivation-related inequalities in flu vaccine uptake amongst primary school children (age 4-9 years) – sensitivity analysis excluding children on the border of age-based vaccine eligibility (i.e.: excluding age 9/10 years).** Results from Cox proportional hazards models adjusted by age are reported as hazard ratios with 95% confidence intervals. The reference groups are D10 (least deprived areas) and age 4 years for each season. The vertical line indicates the onset of the pandemic.

|  | **Flu vaccination season** | | | | |
| --- | --- | --- | --- | --- | --- |
|  | 2018/19 | | 2019/20 | 2020/21 | 2021/22 |
| **IDACI* decile** | |  | |  |  |
| D1 (Most deprived) | 0.61 | | 0.58 | 0.46 | 0.48 |
|  | [0.59,0.63] | | [0.57,0.60] | [0.45,0.47] | [0.47,0.49] |
| D2 | 0.61 | | 0.55 | 0.47 | 0.51 |
|  | [0.60,0.63] | | [0.54,0.57] | [0.46,0.48] | [0.49,0.52] |
| D3 | 0.64 | | 0.55 | 0.48 | 0.54 |
|  | [0.62,0.66] | | [0.53,0.57] | [0.47,0.50] | [0.53,0.56] |
| D4 | 0.77 | | 0.70 | 0.60 | 0.61 |
|  | [0.74,0.79] | | [0.68,0.72] | [0.58,0.62] | [0.59,0.63] |
| D5 | 0.80 | | 0.71 | 0.68 | 0.68 |
|  | [0.77,0.83] | | [0.69,0.74] | [0.66,0.70] | [0.66,0.70] |
| D6 | 0.80 | | 0.73 | 0.70 | 0.75 |
|  | [0.77,0.83] | | [0.70,0.75] | [0.68,0.73] | [0.73,0.78] |
| D7 | 0.95 | | 0.82 | 0.81 | 0.81 |
|  | [0.91,0.98] | | [0.79,0.85] | [0.78,0.83] | [0.79,0.84] |
| D8 | 0.94 | | 0.90 | 0.84 | 0.92 |
|  | [0.91,0.98] | | [0.87,0.93] | [0.81,0.87] | [0.89,0.94] |
| D9 | 1.09 | | 0.96 | 0.99 | 0.96 |
|  | [1.06,1.13] | | [0.93,0.99] | [0.96,1.03] | [0.93,0.99] |
| D10 (Least deprived) | Ref | | Ref | Ref | Ref |
|  | - | | - | - | - |
| **Age (years)** |  | |  |  |  |
| 4 | Ref | | Ref | Ref | Ref |
|  | - | | - | - | - |
| 5 | 1.06 | | 1.10 | 0.92 | 1.16 |
|  | [1.04,1.09] | | [1.07,1.12] | [0.90,0.94] | [1.13,1.18] |
| 6 | 1.04 | | 1.07 | 0.92 | 1.16 |
|  | [1.02,1.07] | | [1.05,1.10] | [0.90,0.95] | [1.14,1.18] |
| 7 | 1.03 | | 1.04 | 0.92 | 1.18 |
|  | [1.01,1.05] | | [1.02,1.07] | [0.90,0.94] | [1.16,1.21] |
| 8 | 1.01 | | 1.02 | 0.91 | 1.16 |
|  | [0.98,1.03] | | [1.00,1.04] | [0.89,0.93] | [1.14,1.19] |
|  |  | |  |  |  |
| **Observations** | 197945 | | 196703 | 196020 | 193266 |

Exponentiated coefficients (hazard ratios); 95% confidence intervals in brackets

* IDACI: Income deprivation affecting children index

D1 – D10: Deprivation deciles 1 - 10
