# Supplementary material for: Evaluating socioeconomic inequalities in influenza vaccine uptake during the COVID-19 pandemic: A cohort study in Greater Manchester, England
Source: PLoS Med. 2023 Sep 26;20(9):e1004289. doi: 10.1371/journal.pmed.1004289 (PMC10522043; doi:10.1371/journal.pmed.1004289)
Supplement: S15 Table — Results from Cox proportional hazards models adjusted by age are reported as hazard ratios with 95% confidence intervals. The reference groups are D10 (least deprived areas) and age 66–69 years for each season. The vertical line indicates the onset of the pandemic. (DOCX) [file pmed.1004289.s018.docx]

**S15 Table. Relative age-adjusted income deprivation-related inequalities in flu vaccine uptake amongst older adults (age 65 years plus) – sensitivity analysis excluding adults on the border of age-based vaccine eligibility (i.e.: excluding age 64/65 years).** Results from Cox proportional hazards models adjusted by age are reported as hazard ratios with 95% confidence intervals. The reference groups are D10 (least deprived areas) and age 66-69 years for each season. The vertical line indicates the onset of the pandemic.

|  | **Flu vaccination season** | | | | | | |
| --- | --- | --- | --- | --- | --- | --- | --- |
|  | 2015/16 | 2016/17 | 2017/18 | 2018/19 | 2019/20 | 2020/21 | 2021/22 |
| **IDAOPI* decile** |  |  |  |  |  |  |  |
| D1 (Most deprived) | 0.80 | 0.81 | 0.78 | 0.76 | 0.77 | 0.69 | 0.63 |
|  | [0.78,0.82] | [0.79,0.82] | [0.77,0.80] | [0.75,0.77] | [0.76,0.79] | [0.68,0.70] | [0.62,0.64] |
| D2 | 0.80 | 0.80 | 0.78 | 0.77 | 0.81 | 0.75 | 0.72 |
|  | [0.79,0.82] | [0.78,0.81] | [0.76,0.79] | [0.76,0.79] | [0.80,0.83] | [0.74,0.76] | [0.70,0.73] |
| D3 | 0.81 | 0.82 | 0.80 | 0.80 | 0.84 | 0.80 | 0.77 |
|  | [0.79,0.83] | [0.80,0.84] | [0.79,0.82] | [0.78,0.82] | [0.82,0.86] | [0.78,0.81] | [0.75,0.78] |
| D4 | 0.86 | 0.87 | 0.85 | 0.85 | 0.87 | 0.83 | 0.81 |
|  | [0.84,0.88] | [0.85,0.88] | [0.83,0.86] | [0.83,0.86] | [0.86,0.89] | [0.82,0.85] | [0.80,0.83] |
| D5 | 0.83 | 0.83 | 0.82 | 0.81 | 0.85 | 0.84 | 0.82 |
|  | [0.81,0.85] | [0.82,0.85] | [0.80,0.84] | [0.79,0.82] | [0.83,0.86] | [0.82,0.85] | [0.81,0.84] |
| D6 | 0.92 | 0.91 | 0.88 | 0.90 | 0.94 | 0.91 | 0.89 |
|  | [0.90,0.94] | [0.89,0.93] | [0.87,0.90] | [0.88,0.91] | [0.92,0.96] | [0.90,0.93] | [0.88,0.91] |
| D7 | 0.90 | 0.89 | 0.88 | 0.88 | 0.93 | 0.90 | 0.88 |
|  | [0.88,0.92] | [0.87,0.91] | [0.86,0.90] | [0.86,0.90] | [0.92,0.95] | [0.88,0.92] | [0.86,0.89] |
| D8 | 0.96 | 0.96 | 0.93 | 0.94 | 0.99 | 0.98 | 0.98 |
|  | [0.94,0.98] | [0.94,0.98] | [0.91,0.95] | [0.92,0.95] | [0.97,1.01] | [0.96,1.00] | [0.96,1.00] |
| D9 | 0.95 | 0.96 | 0.94 | 0.95 | 0.99 | 0.98 | 0.96 |
|  | [0.93,0.97] | [0.94,0.98] | [0.93,0.96] | [0.93,0.96] | [0.97,1.00] | [0.96,1.00] | [0.94,0.98] |
| D10 (Least deprived) | Ref | Ref | Ref | Ref | Ref | Ref | Ref |
|  | - | - | - | - | - | - | - |
| **Age group (years)** |  |  |  |  |  |  |  |
| 66-69 | Ref | Ref | Ref | Ref | Ref | Ref | Ref |
|  | - | - | - | - | - | - | - |
| 70-74 | 1.26 | 1.25 | 1.22 | 1.22 | 1.24 | 1.19 | 1.20 |
|  | [1.25,1.28] | [1.23,1.26] | [1.20,1.23] | [1.21,1.24] | [1.22,1.25] | [1.18,1.20] | [1.19,1.21] |
| 75-79 | 1.40 | 1.40 | 1.36 | 1.39 | 1.38 | 1.31 | 1.34 |
|  | [1.39,1.42] | [1.38,1.42] | [1.34,1.38] | [1.38,1.41] | [1.37,1.40] | [1.29,1.32] | [1.32,1.35] |
| 80+ | 1.23 | 1.26 | 1.25 | 1.29 | 1.29 | 1.21 | 1.29 |
|  | [1.22,1.25] | [1.24,1.27] | [1.23,1.26] | [1.28,1.31] | [1.28,1.30] | [1.20,1.22] | [1.28,1.31] |
|  |  |  |  |  |  |  |  |
| **Observations** | 317243 | 339522 | 362925 | 387130 | 410646 | 420291 | 427114 |

Exponentiated coefficients (hazard ratios); 95% confidence intervals in brackets

* IDAOPI: Income deprivation affecting older people index

D1 – D10: Deprivation deciles 1 - 10
