# Supplementary material for: Evaluating socioeconomic inequalities in influenza vaccine uptake during the COVID-19 pandemic: A cohort study in Greater Manchester, England
Source: PLoS Med. 2023 Sep 26;20(9):e1004289. doi: 10.1371/journal.pmed.1004289 (PMC10522043; doi:10.1371/journal.pmed.1004289)
Supplement: S16 Table — Results from Cox proportional hazards models adjusted by age are reported as hazard ratios with 95% confidence intervals. The reference groups are D10 (least deprived areas), age 0–4 years, and no clinical eligibility for flu vaccination for each season. Deprivation measure is the index of multiple deprivation (IMD). (DOCX) [file pmed.1004289.s019.docx]

**S16 Table. Relative** **age-adjusted deprivation-related inequalities in flu vaccine uptake for 2021/22 vaccination season – sensitivity analysis comparing all-age inequalities across (1) main sample (2) expanded age eligibility for 2021/22 and (3) expanded age eligibility and clinical eligibility.** Results from Cox proportional hazards models adjusted by age are reported as hazard ratios with 95% confidence intervals. The reference groups are D10 (least deprived areas), age 0-4 years, and no clinical eligibility for flu vaccination for each season. Deprivation measure is the index of multiple deprivation (IMD).

|  | **Original** | **Expanded age eligibility** | **Expanded age, plus age 17-49 years who are clinically eligible** |
| --- | --- | --- | --- |
| **Deprivation** |  |  |  |
| D1 (Most deprived) | 0.57 | 0.54 | 0.56 |
|  | [0.56,0.58] | [0.54,0.55] | [0.55,0.56] |
| D2 | 0.60 | 0.57 | 0.58 |
|  | [0.59,0.61] | [0.57,0.58] | [0.58,0.59] |
| D3 | 0.69 | 0.66 | 0.67 |
|  | [0.68,0.70] | [0.65,0.67] | [0.66,0.67] |
| D4 | 0.71 | 0.68 | 0.69 |
|  | [0.69,0.72] | [0.68,0.69] | [0.68,0.70] |
| D5 | 0.79 | 0.76 | 0.76 |
|  | [0.77,0.80] | [0.75,0.77] | [0.75,0.77] |
| D6 | 0.82 | 0.80 | 0.80 |
|  | [0.81,0.84] | [0.79,0.81] | [0.80,0.81] |
| D7 | 0.86 | 0.83 | 0.84 |
|  | [0.85,0.87] | [0.82,0.84] | [0.83,0.85] |
| D8 | 0.88 | 0.87 | 0.87 |
|  | [0.87,0.89] | [0.86,0.88] | [0.86,0.88] |
| D9 | 0.94 | 0.93 | 0.93 |
|  | [0.93,0.96] | [0.92,0.94] | [0.92,0.94] |
| D10 (Least deprived) | Ref | Ref | Ref |
|  | - | - | - |
| **Age group (years)** |  |  |  |
| 0-4 | Ref | Ref | Ref |
|  | - | - | - |
| 5-9 | 1.51 | 1.52 | 1.52 |
|  | [1.49,1.53] | [1.50,1.54] | [1.50,1.55] |
| 10-14 | 1.51 | 1.13 | 1.13 |
|  | [1.48,1.53] | [1.12,1.15] | [1.12,1.15] |
| 15-19 | - | 0.63 | 0.63 |
|  | - | [0.62,0.64] | [0.62,0.64] |
| 20-24 | - | - | 0.37 |
|  | - | - | [0.36,0.38] |
| 25-29 | - | - | 0.43 |
|  | - | - | [0.42,0.44] |
| 30-34 | - | - | 0.50 |
|  | - | - | [0.49,0.51] |
| 35-39 | - | - | 0.61 |
|  | - | - | [0.60,0.63] |
| 40-44 | - | - | 0.72 |
|  | - | - | [0.70,0.74] |
| 45-49 | - | - | 0.87 |
|  | - | - | [0.85,0.89] |
| 50-54 | - | 0.87 | 0.87 |
|  | - | [0.86,0.88] | [0.86,0.88] |
| 55-59 | - | 1.17 | 1.17 |
|  | - | [1.15,1.18] | [1.15,1.19] |
| 60-64 | - | 1.48 | 1.49 |
|  | - | [1.46,1.51] | [1.47,1.51] |
| 65-69 | 2.17 | 2.09 | 2.09 |
|  | [2.14,2.21] | [2.06,2.12] | [2.06,2.12] |
| 70-74 | 2.71 | 2.59 | 2.60 |
|  | [2.67,2.75] | [2.55,2.63] | [2.56,2.64] |
| 75-79 | 2.97 | 2.83 | 2.84 |
|  | [2.93,3.02] | [2.79,2.87] | [2.80,2.89] |
| 80+ | 2.77 | 2.61 | 2.62 |
|  | [2.73,2.81] | [2.57,2.65] | [2.58,2.66] |
| **Clinical eligibility** |  |  |  |
| No | Ref | Ref | Ref |
|  | - | - | - |
| Yes | 1.46 | 1.68 | 1.68 |
|  | [1.44,1.47] | [1.67,1.69] | [1.67,1.69] |
|  |  |  |  |
| **Observations** | 758483 | 1524848 | 1698981 |

Exponentiated coefficients (hazard ratios); 95% confidence intervals in brackets

D1 – D10: Deprivation deciles 1 - 10
