# Supplementary material for: Evaluating socioeconomic inequalities in influenza vaccine uptake during the COVID-19 pandemic: A cohort study in Greater Manchester, England
Source: PLoS Med. 2023 Sep 26;20(9):e1004289. doi: 10.1371/journal.pmed.1004289 (PMC10522043; doi:10.1371/journal.pmed.1004289)
Supplement: S17 Table — Results from Cox proportional hazards models adjusted by age are reported as hazard ratios with 95% confidence intervals. The reference groups are D10 (least deprived areas) and age 2 years for each season. The vertical line indicates the onset of the pandemic. (DOCX) [file pmed.1004289.s020.docx]

**S17 Table. Relative** **age-adjusted income deprivation-related inequalities in flu vaccine uptake amongst pre-school children (age 2-3 years) stratified by sex – Male results.** Results from Cox proportional hazards models adjusted by age are reported as hazard ratios with 95% confidence intervals. The reference groups are D10 (least deprived areas) and age 2 years for each season. The vertical line indicates the onset of the pandemic.

|  | **Flu vaccination season** | | | | | | |
| --- | --- | --- | --- | --- | --- | --- | --- |
|  | 2015/16 | 2016/17 | 2017/18 | 2018/19 | 2019/20 | 2020/21 | 2021/22 |
| **IDACI* decile** |  |  |  |  |  |  |  |
| D1 (Most deprived) | 0.55 | 0.51 | 0.52 | 0.51 | 0.56 | 0.49 | 0.49 |
|  | [0.51,0.59] | [0.48,0.55] | [0.49,0.55] | [0.48,0.55] | [0.52,0.59] | [0.46,0.52] | [0.46,0.53] |
| D2 | 0.60 | 0.53 | 0.52 | 0.52 | 0.53 | 0.47 | 0.51 |
|  | [0.56,0.64] | [0.49,0.56] | [0.48,0.55] | [0.49,0.56] | [0.50,0.57] | [0.44,0.50] | [0.47,0.54] |
| D3 | 0.57 | 0.51 | 0.52 | 0.50 | 0.56 | 0.50 | 0.50 |
|  | [0.53,0.62] | [0.48,0.56] | [0.49,0.57] | [0.46,0.54] | [0.52,0.60] | [0.47,0.54] | [0.47,0.54] |
| D4 | 0.66 | 0.69 | 0.61 | 0.61 | 0.61 | 0.60 | 0.60 |
|  | [0.60,0.71] | [0.64,0.75] | [0.57,0.66] | [0.57,0.66] | [0.57,0.66] | [0.56,0.65] | [0.56,0.65] |
| D5 | 0.72 | 0.69 | 0.65 | 0.66 | 0.71 | 0.63 | 0.61 |
|  | [0.66,0.79] | [0.64,0.76] | [0.60,0.71] | [0.60,0.71] | [0.66,0.78] | [0.58,0.68] | [0.56,0.66] |
| D6 | 0.78 | 0.68 | 0.66 | 0.72 | 0.79 | 0.70 | 0.65 |
|  | [0.71,0.85] | [0.62,0.75] | [0.60,0.72] | [0.66,0.78] | [0.72,0.86] | [0.64,0.76] | [0.60,0.71] |
| D7 | 0.81 | 0.82 | 0.80 | 0.80 | 0.79 | 0.74 | 0.70 |
|  | [0.74,0.89] | [0.76,0.90] | [0.74,0.87] | [0.73,0.87] | [0.73,0.86] | [0.68,0.80] | [0.64,0.76] |
| D8 | 0.80 | 0.83 | 0.86 | 0.85 | 0.85 | 0.84 | 0.83 |
|  | [0.73,0.88] | [0.76,0.91] | [0.79,0.94] | [0.79,0.93] | [0.78,0.92] | [0.78,0.91] | [0.76,0.90] |
| D9 | 0.99 | 1.00 | 0.96 | 0.98 | 1.01 | 0.91 | 0.93 |
|  | [0.91,1.08] | [0.92,1.08] | [0.88,1.03] | [0.91,1.06] | [0.93,1.09] | [0.85,0.98] | [0.86,1.01] |
| D10 (Least deprived) | Ref | Ref | Ref | Ref | Ref | Ref | Ref |
|  | - | - | - | - | - | - | - |
| **Age (years)** |  |  |  |  |  |  |  |
| 2 | Ref | Ref | Ref | Ref | Ref | Ref | Ref |
|  | - | - | - | - | - | - | - |
| 3 | 1.62 | 1.72 | 1.73 | 1.72 | 1.73 | 1.76 | 1.82 |
|  | [1.56,1.68] | [1.66,1.79] | [1.67,1.79] | [1.66,1.79] | [1.67,1.79] | [1.70,1.82] | [1.76,1.89] |
|  |  |  |  |  |  |  |  |
| **Observations** | 40511 | 39928 | 39916 | 39961 | 38982 | 37387 | 36171 |

Exponentiated coefficients (hazard ratios); 95% confidence intervals in brackets

* IDACI: Income deprivation affecting children index

D1 – D10: Deprivation deciles 1 - 10
