# Supplementary material for: Evaluating socioeconomic inequalities in influenza vaccine uptake during the COVID-19 pandemic: A cohort study in Greater Manchester, England
Source: PLoS Med. 2023 Sep 26;20(9):e1004289. doi: 10.1371/journal.pmed.1004289 (PMC10522043; doi:10.1371/journal.pmed.1004289)
Supplement: S19 Table — Results from Cox proportional hazards models adjusted by age are reported as hazard ratios with 95% confidence intervals. The reference groups are D10 (least deprived areas) and age 4 years for each season. The vertical line indicates the onset of the pandemic. (DOCX) [file pmed.1004289.s022.docx]

**S19 Table. Relative** **age-adjusted income deprivation-related inequalities in flu vaccine uptake amongst primary school children (age 4-9 years) stratified by sex – Male results.** Results from Cox proportional hazards models adjusted by age are reported as hazard ratios with 95% confidence intervals. The reference groups are D10 (least deprived areas) and age 4 years for each season. The vertical line indicates the onset of the pandemic.

|  | **Flu vaccination season** | | | | |
| --- | --- | --- | --- | --- | --- |
|  | 2018/19 | | 2019/20 | 2020/21 | 2021/22 |
| **IDACI* decile** | |  | |  |  |
| D1 (Most deprived) | 0.61 | | 0.59 | 0.46 | 0.47 |
|  | [0.59,0.63] | | [0.57,0.61] | [0.44,0.47] | [0.46,0.49] |
| D2 | 0.62 | | 0.56 | 0.47 | 0.50 |
|  | [0.60,0.65] | | [0.54,0.58] | [0.45,0.49] | [0.48,0.51] |
| D3 | 0.65 | | 0.56 | 0.48 | 0.53 |
|  | [0.62,0.67] | | [0.53,0.58] | [0.47,0.50] | [0.51,0.55] |
| D4 | 0.77 | | 0.71 | 0.60 | 0.61 |
|  | [0.73,0.80] | | [0.68,0.74] | [0.57,0.62] | [0.59,0.63] |
| D5 | 0.82 | | 0.73 | 0.69 | 0.68 |
|  | [0.79,0.86] | | [0.70,0.77] | [0.66,0.72] | [0.65,0.70] |
| D6 | 0.81 | | 0.74 | 0.71 | 0.74 |
|  | [0.77,0.85] | | [0.71,0.77] | [0.68,0.74] | [0.71,0.77] |
| D7 | 0.96 | | 0.83 | 0.80 | 0.79 |
|  | [0.91,1.00] | | [0.80,0.87] | [0.76,0.83] | [0.76,0.82] |
| D8 | 0.95 | | 0.90 | 0.83 | 0.89 |
|  | [0.91,1.00] | | [0.86,0.94] | [0.80,0.87] | [0.86,0.93] |
| D9 | 1.10 | | 0.95 | 0.98 | 0.94 |
|  | [1.05,1.15] | | [0.91,0.99] | [0.94,1.02] | [0.90,0.97] |
| D10 (Least deprived) | Ref | | Ref | Ref | Ref |
|  | - | | - | - | - |
| **Age (years)** |  | |  |  |  |
| 4 | Ref | | Ref | Ref | Ref |
|  | - | | - | - | - |
| 5 | 1.09 | | 1.06 | 1.12 | 0.88 |
|  | [1.06,1.13] | | [1.03,1.10] | [1.09,1.16] | [0.86,0.91] |
| 6 | 1.18 | | 1.16 | 1.02 | 1.02 |
|  | [1.14,1.22] | | [1.12,1.19] | [0.99,1.06] | [0.99,1.05] |
| 7 | 1.13 | | 1.15 | 1.03 | 1.01 |
|  | [1.09,1.16] | | [1.11,1.18] | [1.00,1.06] | [0.99,1.04] |
| 8 | 1.10 | | 1.09 | 1.03 | 1.04 |
|  | [1.07,1.14] | | [1.06,1.12] | [1.00,1.06] | [1.01,1.06] |
| 9 | 1.06 | | 1.06 | 1.02 | 1.01 |
|  | [1.03,1.10] | | [1.03,1.09] | [0.99,1.05] | [0.99,1.04] |
|  |  | |  |  |  |
| **Observations** | 121420 | | 121135 | 120814 | 119400 |

Exponentiated coefficients (hazard ratios); 95% confidence intervals in brackets

* IDACI: Income deprivation affecting children index

D1 – D10: Deprivation deciles 1 - 10
