# Supplementary material for: Evaluating socioeconomic inequalities in influenza vaccine uptake during the COVID-19 pandemic: A cohort study in Greater Manchester, England
Source: PLoS Med. 2023 Sep 26;20(9):e1004289. doi: 10.1371/journal.pmed.1004289 (PMC10522043; doi:10.1371/journal.pmed.1004289)
Supplement: S20 Table — Results from Cox proportional hazards models adjusted by age are reported as hazard ratios with 95% confidence intervals. The reference groups are D10 (least deprived areas) and age 4 years for each season. The vertical line indicates the onset of the pandemic. (DOCX) [file pmed.1004289.s023.docx]

**S20 Table. Relative** **age-adjusted income deprivation-related inequalities in flu vaccine uptake amongst primary school children (age 4-9 years) stratified by sex – Female results.** Results from Cox proportional hazards models adjusted by age are reported as hazard ratios with 95% confidence intervals. The reference groups are D10 (least deprived areas) and age 4 years for each season. The vertical line indicates the onset of the pandemic.

|  | **Flu vaccination season** | | | | |
| --- | --- | --- | --- | --- | --- |
|  | 2018/19 | | 2019/20 | 2020/21 | 2021/22 |
| **IDACI* decile** | |  | |  |  |
| D1 (Most deprived) | 0.62 | | 0.59 | 0.47 | 0.50 |
|  | [0.59,0.64] | | [0.57,0.61] | [0.46,0.49] | [0.48,0.51] |
| D2 | 0.61 | | 0.56 | 0.47 | 0.52 |
|  | [0.59,0.64] | | [0.54,0.58] | [0.45,0.48] | [0.51,0.54] |
| D3 | 0.62 | | 0.56 | 0.48 | 0.56 |
|  | [0.60,0.65] | | [0.54,0.58] | [0.46,0.50] | [0.54,0.58] |
| D4 | 0.75 | | 0.70 | 0.62 | 0.61 |
|  | [0.72,0.79] | | [0.67,0.73] | [0.60,0.65] | [0.59,0.63] |
| D5 | 0.76 | | 0.71 | 0.68 | 0.69 |
|  | [0.73,0.80] | | [0.68,0.74] | [0.65,0.71] | [0.66,0.71] |
| D6 | 0.80 | | 0.72 | 0.70 | 0.77 |
|  | [0.76,0.84] | | [0.69,0.76] | [0.67,0.74] | [0.74,0.80] |
| D7 | 0.94 | | 0.82 | 0.82 | 0.84 |
|  | [0.89,0.98] | | [0.78,0.86] | [0.78,0.86] | [0.81,0.87] |
| D8 | 0.93 | | 0.91 | 0.87 | 0.93 |
|  | [0.88,0.97] | | [0.87,0.95] | [0.83,0.91] | [0.90,0.97] |
| D9 | 1.09 | | 0.98 | 1.01 | 0.98 |
|  | [1.05,1.14] | | [0.94,1.02] | [0.97,1.05] | [0.94,1.01] |
| D10 (Least deprived) | Ref | | Ref | Ref | Ref |
|  | - | | - | - | - |
| **Age (years)** |  | |  |  |  |
| 4 | Ref | | Ref | Ref | Ref |
|  | - | | - | - | - |
| 5 | 1.00 | | 1.00 | 1.10 | 0.87 |
|  | [0.97,1.04] | | [0.97,1.03] | [1.06,1.13] | [0.84,0.89] |
| 6 | 1.05 | | 1.10 | 1.02 | 1.00 |
|  | [1.02,1.09] | | [1.07,1.14] | [0.99,1.05] | [0.98,1.03] |
| 7 | 1.06 | | 1.07 | 1.02 | 1.02 |
|  | [1.02,1.09] | | [1.03,1.10] | [0.99,1.05] | [0.99,1.05] |
| 8 | 1.05 | | 1.06 | 1.02 | 1.04 |
|  | [1.02,1.09] | | [1.02,1.09] | [0.98,1.05] | [1.01,1.06] |
| 9 | 1.05 | | 1.04 | 1.00 | 1.02 |
|  | [1.01,1.08] | | [1.01,1.08] | [0.97,1.03] | [0.99,1.05] |
|  |  | |  |  |  |
| **Observations** | 115963 | | 115512 | 114906 | 113872 |

Exponentiated coefficients (hazard ratios); 95% confidence intervals in brackets

* IDACI: Income deprivation affecting children index

D1 – D10: Deprivation deciles 1 - 10
