# Supplementary material for: Evaluating socioeconomic inequalities in influenza vaccine uptake during the COVID-19 pandemic: A cohort study in Greater Manchester, England
Source: PLoS Med. 2023 Sep 26;20(9):e1004289. doi: 10.1371/journal.pmed.1004289 (PMC10522043; doi:10.1371/journal.pmed.1004289)
Supplement: S21 Table — Results from Cox proportional hazards models adjusted by age are reported as hazard ratios with 95% confidence intervals. The reference groups are D10 (least deprived areas) and age 65–69 years for each season. The vertical line indicates the onset of the pandemic. (DOCX) [file pmed.1004289.s024.docx]

**S21 Table. Relative** **age-adjusted income deprivation-related inequalities in flu vaccine uptake amongst older adults (age 65 years plus) stratified by sex – Male results.** Results from Cox proportional hazards models adjusted by age are reported as hazard ratios with 95% confidence intervals. The reference groups are D10 (least deprived areas) and age 65-69 years for each season. The vertical line indicates the onset of the pandemic.

|  | **Flu vaccination season** | | | | | | |
| --- | --- | --- | --- | --- | --- | --- | --- |
|  | 2015/16 | 2016/17 | 2017/18 | 2018/19 | 2019/20 | 2020/21 | 2021/22 |
| **IDAOPI* decile** |  |  |  |  |  |  |  |
| D1 (Most deprived) | 0.80 | 0.81 | 0.79 | 0.77 | 0.77 | 0.68 | 0.62 |
|  | [0.78,0.82] | [0.79,0.83] | [0.77,0.82] | [0.75,0.79] | [0.76,0.79] | [0.67,0.70] | [0.61,0.64] |
| D2 | 0.80 | 0.79 | 0.78 | 0.77 | 0.81 | 0.74 | 0.70 |
|  | [0.78,0.83] | [0.77,0.82] | [0.76,0.80] | [0.75,0.79] | [0.79,0.83] | [0.72,0.76] | [0.68,0.72] |
| D3 | 0.82 | 0.82 | 0.81 | 0.80 | 0.83 | 0.79 | 0.75 |
|  | [0.79,0.84] | [0.79,0.84] | [0.79,0.83] | [0.78,0.82] | [0.81,0.86] | [0.77,0.81] | [0.73,0.77] |
| D4 | 0.85 | 0.86 | 0.84 | 0.83 | 0.87 | 0.82 | 0.80 |
|  | [0.82,0.88] | [0.84,0.89] | [0.81,0.86] | [0.81,0.86] | [0.84,0.89] | [0.79,0.84] | [0.78,0.82] |
| D5 | 0.83 | 0.83 | 0.81 | 0.80 | 0.84 | 0.82 | 0.80 |
|  | [0.80,0.86] | [0.80,0.85] | [0.79,0.83] | [0.77,0.82] | [0.82,0.87] | [0.80,0.85] | [0.78,0.83] |
| D6 | 0.91 | 0.91 | 0.88 | 0.89 | 0.94 | 0.91 | 0.88 |
|  | [0.88,0.94] | [0.88,0.93] | [0.86,0.91] | [0.87,0.92] | [0.91,0.96] | [0.89,0.94] | [0.86,0.90] |
| D7 | 0.90 | 0.89 | 0.88 | 0.87 | 0.93 | 0.90 | 0.87 |
|  | [0.87,0.93] | [0.86,0.91] | [0.86,0.91] | [0.85,0.90] | [0.91,0.96] | [0.87,0.92] | [0.85,0.89] |
| D8 | 0.96 | 0.96 | 0.94 | 0.94 | 0.99 | 0.99 | 0.98 |
|  | [0.93,0.99] | [0.94,0.99] | [0.91,0.96] | [0.91,0.96] | [0.96,1.02] | [0.96,1.01] | [0.96,1.00] |
| D9 | 0.96 | 0.96 | 0.94 | 0.94 | 0.99 | 0.98 | 0.96 |
|  | [0.93,0.99] | [0.93,0.99] | [0.92,0.97] | [0.92,0.97] | [0.96,1.01] | [0.96,1.01] | [0.94,0.98] |
| D10 (Least deprived) | Ref | Ref | Ref | Ref | Ref | Ref | Ref |
|  | - | - | - | - | - | - | - |
| **Age group (years)** |  |  |  |  |  |  |  |
| 65-69 | Ref | Ref | Ref | Ref | Ref | Ref | Ref |
|  | - | - | - | - | - | - | - |
| 70-74 | 1.41 | 1.40 | 1.37 | 1.37 | 1.44 | 1.34 | 1.31 |
|  | [1.39,1.44] | [1.38,1.43] | [1.35,1.39] | [1.35,1.39] | [1.41,1.46] | [1.32,1.36] | [1.29,1.33] |
| 75-79 | 1.61 | 1.60 | 1.56 | 1.59 | 1.63 | 1.51 | 1.48 |
|  | [1.58,1.64] | [1.58,1.63] | [1.53,1.59] | [1.56,1.61] | [1.60,1.65] | [1.48,1.53] | [1.46,1.50] |
| 80+ | 1.48 | 1.50 | 1.49 | 1.54 | 1.58 | 1.45 | 1.49 |
|  | [1.45,1.50] | [1.47,1.52] | [1.46,1.51] | [1.51,1.57] | [1.56,1.61] | [1.43,1.47] | [1.47,1.51] |
|  |  |  |  |  |  |  |  |
| **Observations** | 154840 | 166480 | 178780 | 190987 | 203456 | 209443 | 214009 |

Exponentiated coefficients (hazard ratios); 95% confidence intervals in brackets

* IDAOPI: Income deprivation affecting older people index

D1 – D10: Deprivation deciles 1 - 10
