# Supplementary material for: Evaluating socioeconomic inequalities in influenza vaccine uptake during the COVID-19 pandemic: A cohort study in Greater Manchester, England
Source: PLoS Med. 2023 Sep 26;20(9):e1004289. doi: 10.1371/journal.pmed.1004289 (PMC10522043; doi:10.1371/journal.pmed.1004289)
Supplement: S23 Table — Age-standardised vaccine uptake (%) with 95% confidence intervals. The vertical line indicates the onset of the pandemic. Results also shown in Fig 3 in the main text. (DOCX) [file pmed.1004289.s026.docx]

**S23 Table. Absolute age-adjusted income deprivation-related inequalities in flu vaccine uptake over time.** Age-standardised vaccine uptake (%) with 95% confidence intervals. The vertical line indicates the onset of the pandemic. Results also shown in Figure 3 in the main text.

|  | **Flu vaccination season** | | | | | | |
| --- | --- | --- | --- | --- | --- | --- | --- |
|  | 2015/16 | 2016/17 | 2017/18 | 2018/19 | 2019/20 | 2020/21 | 2021/22 |
| **Age 2-3 years (IDACI^1^)** |  |  |  |  |  |  |  |
| D1 (Most deprived) | 22.38 | 25.47 | 28.09 | 26.93 | 29.94 | 33.20 | 29.51 |
|  | [21.78,22.96] | [24.85,26.08] | [27.45,28.73] | [26.30,27.55] | [29.28,30.61] | [32.52,33.91] | [28.83,30.19] |
| D10 (Least deprived) | 37.58 | 42.23 | 45.57 | 44.91 | 47.53 | 56.25 | 49.68 |
|  | [36.39,38.75] | [41.02,43.43] | [44.36,46.79] | [43.70,46.11] | [46.27,48.78] | [54.98,57.52] | [48.38,50.98] |
|  |  |  |  |  |  |  |  |
| **Age 4-9 years (IDACI^1^)** |  |  |  |  |  |  |  |
| D1 (Most deprived) | - | - | - | 32.13 | 34.59 | 33.48 | 42.41 |
|  |  |  |  | [31.75,32.52] | [34.20,34.99] | [33.09,33.88] | [42,42.82] |
| D10 (Least deprived) | - | - | - | 46.77 | 51.48 | 56.86 | 68.33 |
|  |  |  |  | [46.07,47.48] | [50.77,52.18] | [56.16,57.57] | [67.66,69.01] |
|  |  |  |  |  |  |  |  |
| **Age 65 years plus (IDAOPI^2^)** |  |  |  |  |  |  |  |
| D1 (Most deprived) | 57.16 | 58.11 | 59.57 | 58.18 | 60.13 | 65.52 | 64.90 |
|  | [56.73,57.62] | [57.69,58.53] | [59.16,59.98] | [57.79,58.59] | [59.75,60.50] | [65.16,65.90] | [64.52,65.26] |
| D10 (Least deprived) | 64.97 | 65.41 | 67.76 | 67.86 | 68.84 | 77.76 | 80.41 |
|  | [64.33,65.59] | [64.81,66.02] | [67.16,68.33] | [67.29,68.44] | [68.29,69.40] | [77.26,78.25] | [79.94,80.88] |

Age-standardised percentage vaccine uptake; 95% confidence intervals in brackets

^1^ IDACI: Income deprivation affecting children index

^2^ IDAOPI: Income deprivation affecting older people index

D1 – D10: Deprivation deciles 1 - 10
