# Supplementary material for: Evaluating socioeconomic inequalities in influenza vaccine uptake during the COVID-19 pandemic: A cohort study in Greater Manchester, England
Source: PLoS Med. 2023 Sep 26;20(9):e1004289. doi: 10.1371/journal.pmed.1004289 (PMC10522043; doi:10.1371/journal.pmed.1004289)
Supplement: S24 Table — Age-adjusted estimated difference in vaccine uptake (percentage points) between the least and most income-deprived areas. Estimates shown with 95% confidence intervals. The vertical line indicates the onset of the pandemic. Results also shown in Fig 4 in the main text. (DOCX) [file pmed.1004289.s027.docx]

**S24 Table. Slope index of inequality (SII) by income deprivation for age-adjusted flu vaccine uptake inequalities over time.** Age-adjusted estimated difference in vaccine uptake (percentage points) between the least and most income-deprived areas. Estimates shown with 95% confidence intervals. The vertical line indicates the onset of the pandemic. Results also shown in Figure 4 in the main text.

|  | **Flu vaccination season** | | | | | | |
| --- | --- | --- | --- | --- | --- | --- | --- |
|  | 2015/16 | 2016/17 | 2017/18 | 2018/19 | 2019/20 | 2020/21 | 2021/22 |
|  |  |  |  |  |  |  |  |
| **Age 2-3 years (IDACI^1^)** | 15.59 | 18.42 | 19.37 | 20.07 | 19.82 | 25.25 | 20.86 |
|  | [14.52,16.67] | [17.29,19.53] | [18.23,20.51] | [18.94,21.20] | [18.66,20.98] | [24.04,26.45] | [19.65,22.05] |
|  |  |  |  |  |  |  |  |
| **Age 4-9 years (IDACI^1^)** |  |  |  | 19.12 | 19.23 | 28.09 | 30.27 |
|  |  |  |  | [18.45,19.79] | [18.54,19.91] | [27.41,28.77] | [29.58,30.95] |
|  |  |  |  |  |  |  |  |
| **Age 65 years plus (IDAOPI^2^)** | 8.48 | 7.89 | 8.47 | 10.13 | 9.83 | 13.87 | 16.91 |
|  | [7.91,9.04] | [7.34,8.43] | [7.94,8.99] | [9.62,10.64] | [9.30,10.32] | [13.41,14.33] | [16.46,17.36] |
|  |  |  |  |  |  |  |  |

Slope index of inequality (SII); 95% confidence intervals in brackets

^1^ IDACI: Income deprivation affecting children index

^2^ IDAOPI: Income deprivation affecting older people index
