# Supplementary material for: Evaluating socioeconomic inequalities in influenza vaccine uptake during the COVID-19 pandemic: A cohort study in Greater Manchester, England
Source: PLoS Med. 2023 Sep 26;20(9):e1004289. doi: 10.1371/journal.pmed.1004289 (PMC10522043; doi:10.1371/journal.pmed.1004289)
Supplement: Attachment — Submitted filename: Review PMED-D-23-00344_R1.docx [file pmed.1004289.s028.docx]

**General feedback**

This paper focuses on the socio-economic inequalities associated with uptake of influenza vaccination in the Greater Manchester Region, UK. The authors conducted their statistical analyses with data spanning several subsequent years of flu seasons, the most recent two taking place during the COVID19 pandemic (2020-21 and 2021-22). They used income deprivation as the primary exposure and study three separate age groups: preschool, primary and older (>=65 years) individuals. Their results show that the least deprived populations have a higher uptake of influenza vaccination compared to the most deprived groups across the years. This gap increased in all age groups after the onset of the pandemic due to an increased uptake in the least deprived group compared to the most deprived group. The authors conducted several sensitivity analyses.

This study makes use of a rich dataset and presents useful findings to advance knowledge on inherent inequalities in access to influenza vaccination. The paper is clearly presented and written, and includes a variety of illustrative figures and detailed tables.

**Specific comments to each section of the paper**

1. Abstract
   a. It is not clear what the uptake gap represents. This becomes clear when reading the Results section (absolute difference in % uptake). Can you update the abstract with a clearer definition of ‘uptake gap’?
2. Methods
   a. “We allowed the sample to vary for each vaccination season based on eligibility (age) and deaths (Figure S1).” : This sentence would read better as “The sample varied for each vaccination season (…)”, unless the authors can explain more their choice of wording.
   b. Main statistical analysis: Why was a time to event outcome (time until vaccination) analysis preferred over a binary outcome (vaccination yes/no) analysis?
   c. What baseline date what used to calculate the time to event outcome? Was it the beginning of the flu season for each year or a specific day of the year?
3. Results
a. The Figures in the Results section are great illustrations to display the findings, especially Figures 1 and 2.
b. “To compare trends in inequalities over time, we restricted our sample to groups that were consistently eligible for flu vaccination over multiple seasons, focusing on pre-school children, a subset of primary school children over a subset of seasons, and older adults aged 65 plus.” It’s unclear what this sentence means for each age group. For the older age group, does it mean the sample is restricted to individuals who are >=65years old in 2015/16? If so, I don’t know how this would translate for the 2 younger age groups. Or does it mean the sample is restricted to seasons when each age group is eligible for vaccination? In this case the sentence could be reworded: pre-school and >=65 are included for all seasons, and primary school are included for 2018/19 to 2021/22.
c. Tables S1-S13: Should the label say “adjusted by income-deprivation and by age”?
d. Table S5 seem identical to Table S2, except for the number of observations. Is this because there are very few additional observations included in S5?
e. Comparing Table S9 to S3: the hazard ratios for D1 are slightly lower in 20/21 and 21/22, so there is a less sharper increases in inequality after the onset of the pandemic. Could this warrant the results been sensitive to the choice of deprivation measure for the older age group?
f. “However, amongst pre-school children, excluding those who may have been in primary school resulted in estimates of inequality that were relatively consistent across all vaccination seasons (Table S10)”: what are the changes between S1 and S10 related to this comment?
g. “Inclusion of people clinically eligible for flu vaccination and those eligible due to expanded 2021/22 age criteria indicated inequalities were similar compared to the restricted main study population (Table S13).”
h. “Inclusion of people (…) eligible due to expanded 2021/22 age criteria indicated inequalities were similar compared to the restricted main study population (Table S13). Is this referring to the comparison of Table S13 0.56 (D1) to Table S3 0.63 (D1) ?
