# Supplementary material for: Evaluating socioeconomic inequalities in influenza vaccine uptake during the COVID-19 pandemic: A cohort study in Greater Manchester, England
Source: PLoS Med. 2023 Sep 26;20(9):e1004289. doi: 10.1371/journal.pmed.1004289 (PMC10522043; doi:10.1371/journal.pmed.1004289)
Supplement: Attachment — Submitted filename: Response_to_reviewers.docx [file pmed.1004289.s029.docx]

We are grateful to all three reviewers and the editors for their careful review of the manuscript and thoughtful comments. We have responded to each comment in turn as outlined below and believe that these helpful comments have guided substantial improvements to the manuscript.

**Requests from the editors:**

GENERAL

Please ensure consistency in your number format and revise your manuscript accordingly including the Supplementary Material (51·4% or 51.4%).

We have edited throughout for consistency (format: 51.4%)

For all observational studies, in the manuscript text, please indicate: (1) the specific hypotheses you intended to test, (2) the analytical methods by which you planned to test them, (3) the analyses you actually performed, and (4) when reported analyses differ from those that were planned, transparent explanations for differences that affect the reliability of the study's results. If a reported analysis was performed based on an interesting but unanticipated pattern in the data, please be clear that the analysis was data-driven.

Did your study have a prospective protocol or analysis plan? Please state this (either way) early in the Methods section.

a) If a prospective analysis plan (from your funding proposal, IRB or other ethics committee submission, study protocol, or other planning document written before analyzing the data) was used in designing the study, please include the relevant prospectively written document with your revised manuscript as a Supporting Information file to be published alongside your study, and cite it in the Methods section. A legend for this file should be included at the end of your manuscript.

b) If no such document exists, please make sure that the Methods section transparently describes when analyses were planned, and when/why any data-driven changes to analyses took place.

c) In either case, changes in the analysis-- including those made in response to peer review comments-- should be identified as such in the Methods section of the paper, with rationale.

Responding to these comments together, we have added the following to the methods: “Analysis plan and changes: The hypothesis for this study was that changes associated with the onset of the COVID-19 pandemic may have widened socioeconomic inequalities in flu vaccine uptake. There was no formal prospective protocol but in advance of any analysis, we planned to test this by using area-level IMD decile as the socioeconomic measure and investigating both relative inequalities using age-adjusted Cox proportional hazards models for each flu vaccination season, and absolute inequalities using age-standardised uptake. We later added the SII analysis following feedback from colleagues. At the suggestion of one of the reviewers, we added supplementary analysis stratified by sex, and added results unadjusted by age at the request of the editor. We initially planned to focus only on older adults, as we were not aware data for under 16s was available in this dataset. However, after discussion with data engineers, we also included children who were eligible for flu vaccination. There were no data-driven changes in our approach to the analysis.” (L154)

FINACIAL DISCLOSURE

It appears that one or more study authors is affiliated with one or more of the agencies that funded the study. Thus, the statement “The funders had no role in study design, data collection and analysis, decision to publish, or preparation of the manuscript” does not apply. Please revise the Financial Disclosure accordingly, as in "[Author name] is [author's role] at [funding agency]. The funders had no other role in study design…..”

We have updated the financial disclosure as requested: “Matt Sutton is an NIHR Senior Investigator. The funders had no other role in study design, data collection and analysis, decision to publish, or preparation of the manuscript.

COMPETING INTEREST STATEMENT

All authors must declare their relevant competing interests per the PLOS policy, which can be seen here:

<https://journals.plos.org/plosmedicine/s/competing-interests>

For authors with ties to industry, please indicate whether any of the interests has a financial stake in the results of the current study.

We have declared no competing interests.

ABSTRACT

PLOS Medicine requests that main results are quantified with 95% CIs as well as p values. Please include. When reporting p values please report as p<0.001 and where higher as the exact p value p=0.002, for example. For the purposes of transparent data reporting, if not including the aforementioned please clearly state the reasons why not.

We have provided 95% CIs throughout and have chosen not to report p-values for clarity. Because models were run individually for each vaccination season, p-values would refer to statistical significance for each level of income deprivation within a season. However, most comparisons made within the text are for the same level of deprivation, comparing between vaccination seasons. Reported 95% CIs allow readers to interpret how meaningful any differences are, and adding a p-value for the significance of a different comparison may introduce confusion.

We have added the following to the methods to justify this decision: “We reported results as hazard ratios (HR) with 95% confidence intervals. For clarity, we did not report p-values as these refer to within-season comparisons while most comparisons made in the text are between vaccination seasons.” (L168)

Please include any important dependent variables that are adjusted for in the analyses.

We have added “age-adjusted” (L40)

Abstract Background: Provide the context of why the study is important. The final sentence should clearly state the study question.

We have edited the final sentence of the background section as follows: “However, no existing studies have investigated the consequences for inequalities in flu vaccine uptake, so we aimed to investigate whether socioeconomic inequalities in flu vaccine uptake have widened since the onset of the COVID-19 pandemic.” (L33)

Abstract Methods and Findings:

* Please include the number of participants.

We have added this “The sample population varied between 418,790 (2015-16) and 758,483 (2021/22) across each vaccination season.” (L39)

* In the last sentence of the Abstract Methods and Findings section, please describe the main limitation(s) of the study's methodology.

We have now added: “A limitation of our approach is the use of a local dataset, which may limit generalisability to other geographical settings.” (L52)

Please define all abbreviations used for statistical reporting at first use.

We have done this

Line 37 – Please define NHS at first in the abstract.

Added: “National Health Service (NHS)” (L38)

Line 42 – Throughout, suggest reporting statistical information as follows to improve clarity for the reader “22% (95% CI [13%,28%]; p</=)”. Please amend throughout the abstract and main manuscript.

Please note the use of commas to separate upper and lower bounds, as opposed to hyphens as these can be confused with reporting of negative values.

We have amended throughout as suggested, but without addition of p-values (discussed above). For example: “8.48 (95% CI [7.91,9.04])” (L45)

AUTHOR SUMMARY

At this stage, we ask that you include a short, non-technical Author Summary of your research to make findings accessible to a wide audience that includes both scientists and non-scientists. The Author Summary should immediately follow the Abstract in your revised manuscript. This text is subject to editorial change and should be distinct from the scientific abstract. Please see our author guidelines for more information: <https://journals.plos.org/plosmedicine/s/revising-your-manuscript#loc-author-summary>.

The summary should include 2-3 single sentence, individual bullet points under each of the questions.

It may be helpful to review currently published articles for examples which can be found on our website here <https://journals.plos.org/plosmedicine/>

We have added an author summary at the end of the manuscript (L449):

**Why was this study done?**

- There are known socioeconomic inequalities in flu vaccine uptake, with lower uptake in more deprived neighbourhoods compared to less deprived areas.
- During the COVID-19 pandemic, there were many changes that may have affected flu vaccine uptake, including changes in flu vaccine eligibility and delivery, extensive vaccine messaging and debate linked to the COVID-19 mass-vaccination campaign, and widespread disruption to healthcare services.
- However, no existing studies had analysed whether the COVID-19 pandemic was linked to changes in socioeconomic inequalities in flu vaccine uptake.

**What did the authors do and find?**

- We used health records for the population of Greater Manchester, a large metropolitan region in the North West of England, to look at changes in socioeconomic inequalities in flu vaccine uptake before and during the COVID-19 pandemic.
- We focused on young children and older adults, as these age groups are at higher risk of severe outcomes from flu infection so are eligible for annual flu vaccination provided free at the point of service.
- We found that the difference in uptake between the least and most deprived areas increased markedly during the pandemic years (2020/21 and 2021/22), with the greatest increase in inequalities amongst older adults.

**What do these findings mean?**

- The COVID-19 pandemic led to increased socioeconomic inequalities in flu vaccine uptake, likely due to changes in demand for and access to flu vaccination.
- While this study focused on Greater Manchester specifically, this is a relatively diverse region, and we expect our conclusions to be broadly applicable to the UK population.
- It will be important to better understand the causes of these increased inequalities, and to investigate whether inequalities have also increased across other sociodemographic factors and for uptake of other routine vaccinations.

INTRODUCTION

Please address the potential importance of your study. Indicate whether your study is novel and how you determined that. If there has been a systematic review of the evidence related to your study (or you have conducted one), please refer to and reference that review and indicate whether it supports the need for your study.

We have added the following to make clear the importance of the study: “Flu vaccination provides protection against severe clinical flu presentation [2,3], so vaccine uptake is an important determinant of outcomes from flu infection.” (L62)

And have added further information about the novelty: “To the best of our knowledge, there are no published studies or grey literature that investigate whether inequalities in flu vaccine uptake have changed since the COVID-19 pandemic.” (L88)

Considering a global readership, please include a short statement about the role and function of the NHS.

We have added the following statement: “In England, the NHS provides healthcare free at the point of service, and this includes annual flu vaccination for groups at higher risk of severe outcomes from infection (young children and adults who are aged over 65, have certain pre-existing health conditions, or are pregnant [3].)” (L64)

Please elaborate on the choice of greater Manchester as the study setting, the wider sociodemographic and socioeconomic setting associated with the area and provide justification why the study setting is suitable for general validity of the results (outside of Manchester).

Thank you for this suggestion. We have added the following information and justification in the introduction: “We use population-wide data from a large metropolitan area (Greater Manchester, population 2.8M) to analyse socioeconomic inequalities in flu vaccine uptake before and during the COVID-19 pandemic. Although Greater Manchester is mostly a major urban conurbation, it also encompasses some smaller urban towns as well as some rural towns and villages [20]. Greater Manchester is also a socioeconomically diverse area, including some of the least deprived neighbourhoods nationally, despite overall levels of deprivation higher than the national average [21]. For these reasons, we believe Greater Manchester is a useful setting to study changes in socioeconomic inequalities in vaccine uptake.” (L89)

METHODS AND RESULTS

PLOS Medicine requests that the main outcomes are quantified with p values as well as 95% CIs. Please report p values as p<0.001 and where higher as p=0.002, for example. If not including p values, to help facilitate transparent data reporting, please clearly state the reasons why not. When a p value is given, please specify the statistical test used to determine it.

Suggest reporting statistical information as detailed above – see under ABSTRACT

Please see above for the discussion about inclusion of p-values.

Please present numerators and denominators for percentages, at least in the Tables.

We reported numerators in the main text and provided denominators in the tables.

Line 90 – Please define GP in the Methods.

Added: “general practice (GP) surgery” (L102)

Line 109 – Please change ‘focus’ to ‘focused’ and ensure to keep the tense consistent throughout the manuscript.

Thanks for this edit, we have done as suggested.

Line 159 – Please define NIHR in the Methods.

Added: “National Institute of Health and Care Research (NIHR)” (L191)

Please include in the figure caption of Figure 2 and 3 the age range of pre-school children, primary school children, and older adults.

We have edited the figure captions as suggested. For example Fig 2: “Relative age-adjusted income deprivation-related inequalities in flu vaccine uptake. Results from Cox proportional hazards models adjusted by age are plotted as hazard ratios with 95% confidence intervals. The reference group is D10 (least deprived areas) for each season. The vertical dashed grey line indicates the onset of the pandemic. (A) Inequalities amongst pre-school children (age 2-3), estimated using the income deprivation affecting children index (IDACI) (B) Inequalities amongst primary school children (age 4-9), estimated using IDACI (C) Inequalities amongst older adults (age 65 plus), estimated using income deprivation affecting older people index (IDAOPI).”

In Figure 2 and 3, please show the axis beginning at zero. If this is not possible, please show a break in the axis.

This is not possible for Fig 2 because it is a log scale, so we have added a break to indicate this. We have edited Fig 3 so the axis now begins at 0.

In Figure 4, please define g IDACI and IDAOPI and include in the figure caption the age range of pre-school children, primary school children, and older adults.

We have added this as in the example for Fig 2 above

DISCUSSION

Please present and organize the Discussion as follows: a short, clear summary of the article's findings; what the study adds to existing research and where and why the results may differ from previous research; strengths and limitations of the study; implications and next steps for research, clinical practice, and/or public policy; one-paragraph conclusion.

We have re-ordered the discussions sections as requested.

Lines 308-309: Please include a reference for the following statement “Given that older adults are more vulnerable than children to severe COVID-19, […]”.

We have added the following citation: “Given that older adults are more vulnerable than children to severe COVID-19 [29]” (L323)

Ref 29: Office for National Statistics. Deaths involving coronavirus (COVID-19) by age. In: Coronavirus (COVID-19) latest insights [Internet]. [cited 12 May 2023]. Available: https://www.ons.gov.uk/peoplepopulationandcommunity/healthandsocialcare/conditionsanddiseases/articles/coronaviruscovid19latestinsights/deaths#deaths-by-age

FIGURES AND TABLES

Please ensure to define the abbreviations used in your figures and tables (e.g. in Table 1 add “IQR = interquartile range” underneath the table)

We have added all abbreviations as suggested.

Please provide titles and legends for all figures (including those in Supporting Information files).

We have added these at the end of the manuscript (L600).

For example: “S1 Table. Relative age-adjusted income deprivation-related inequalities in flu vaccine uptake amongst pre-school children (age 2-3). Results from Cox proportional hazards models adjusted by age are reported as hazard ratios with 95% confidence intervals. The reference groups are D10 (least deprived areas) and age 2 for each season. The vertical line indicates the onset of the pandemic. Results also shown in Figure 2 in the main text.”

Please indicate in figure captions whether analyses are adjusted or unadjusted and where adjusted please detail the factors adjusted for.

Where adjusted analyses are presented, to help facilitate transparent data reporting please also unadjusted analyses for comparison.

We have added this information to all figure captions (as in the example for S1 Table above). We have also added three new supplementary tables showing results unadjusted by age (S2 Table, S4 Table, S6 Table) and clarified in the analysis plan section that this was a change requested by the editors: “and added results unadjusted by age at the request of the editor” (L160)

Where 95% CIs are reported PLOS Medicine also requires that p values are reported. Please include in the tables and figures. Please report p as <0.001 and where higher as p=0.002, for example Please replace the use of hyphens with commas to separate upper and lower CI bounds as these can be confused with negative values.

We have replaced the hyphens with commas throughout.

Please consider the use of a color palate suitable for those with color blindness.

Thanks for this suggestion. We have now checked all figures and changed the colour scheme for figures 2, 3 & 4 to be suitable.

In the Supplementary Material, in the Table of contents, please change “STROBE Statemen” to “STROBE Statement”.

We have corrected this.

In the Supplementary Tables, please explain in the caption the values (IDACI decile D10 or age) used as reference points (in the table shown as ‘REF’).

We have edited all supplementary table captions as suggested and any abbreviations are explained in the footnotes. For example: “S1 Table. Relative age-adjusted income deprivation-related inequalities in flu vaccine uptake amongst pre-school children (age 2-3). Results from Cox proportional hazards models adjusted by age are reported as hazard ratios with 95% confidence intervals. The reference groups are D10 (least deprived areas) and age 2 for each season. The vertical line indicates the onset of the pandemic. Results also shown in Figure 2 in the main text.”

REFERENCES

Please cite your Supporting Information as outlined here: <https://journals.plos.org/plosmedicine/s/supporting-information>

We have now saved each supporting information file individually and followed the PLOS guidelines for citing supporting information.

Please cite the reference numbers in square brackets (e.g., “We used the techniques developed by our colleagues [19] to analyze the data”). Citations should be preceding punctuation.

PLOS uses the numbered citation (citation-sequence) method and first six authors, et al.

We have used the PLOS citation style.

**Reviewer #1: Alex McConnachie, Statistical Review**

Watkinson et al present data from a General Practice database covering the Manchester area, looking at socioeconomic differences in flu vaccine uptake from 2015/16 to 2021/22. This review looks at the use of statistics in the paper.

These are generally very good. The data are clearly described and presented. Cox models are used to analyse time to vaccination, and age-standardised vaccination rates are analysed using the slope index of inequality. A number of sensitivity analyses are presented to assess the robustness of the findings. The results appear to be interpreted appropriately. My comments are relatively minor.

In the abstract, the statement "…vaccine uptake increased across all socioeconomic groups during the pandemic" is not supported by any of the data reported at that point.

We have now edited to “Although vaccine uptake increased during the pandemic, disproportionately larger increases in uptake in less deprived areas created wider inequalities in all age groups.” (L49)

The section on covariates makes no mention of gender. Looking at Table 1, this data is available, and looks virtually complete. Why was this not considered, at least in the adult cohort? The other obvious factor of interest would be ethnicity, though I assume the data here is less reliable. Should this at least be noted as a limitation?

Thank you for this interesting suggestion. We have now additionally stratified by gender and repeated the main analysis. We have updated the methods, results, and discussion as follows:

Methods: “At the suggestion of one of the reviewers, we added supplementary analysis stratified by sex” (L159) & “In addition, we re-estimated the main results stratifying by sex (male or female). (L185)

Results: Added supplementary tables & “Stratification by sex (male or female) indicated that socioeconomic inequalities in flu vaccine uptake did not vary substantially by sex across any age group or vaccination season (S17 Table – S22 Table).” (L262)

We have also added the lack of analysis by ethnic group as a limitation in the discussion: “Although we did not find evidence that socioeconomic inequalities in flu vaccine uptake varied by sex across any age group, there are likely other intersecting axes of health inequality that may contribute to overall inequalities in flu vaccine uptake. For example, a limitation of our analysis is that we did not explore inequalities by ethnic group, which is a known determinant of both flu vaccine and COVID-19 vaccine uptake in this study population [24]. Given that structural racism results in people from ethnic minority backgrounds being over-represented in more deprived areas [34], our results raise the possibility that ethnic inequalities in flu vaccine uptake may also have widened, which will be an important area for future research.” (L363)

And “Further research is needed to understand whether inequalities along other sociodemographic axes of inequality and for other routine vaccinations have also widened.” (L419)

As ever with Cox models, there is the question of the proportional hazards assumption. Was this checked? My guess, with a dataset of this size, there may be some evidence of non-PH, but as long as it is not too severe, then a PH model should be OK.

Thank you for raising this important point. As expected, there is some evidence of non-PH in this large dataset, but we checked log(−log[survival]) versus log(time) plots and believe these indicate the PH assumption is reasonable. We have now included these in the manuscript with the following changes:

Methods: “We confirmed that the proportional hazards assumption was reasonable by visual inspection of log(−log[survival]) versus log(time) plots (S2 Fig).” (L170)

Figures: We have added a new S2 Figure showing plots for each age group and each vaccination season.

In Table 1, age is reported as median and quartiles, which is fine, but why report to one decimal place? I assume the data are integers. Also, on a stylistic note, perhaps the percentages for deaths in the child cohorts could be reported as "<0.1%" and ">99.9%"?

Thank you, we have made these changes as suggested (Table 1)

In the discussion, line 342 talks of "this winter". Someone reading this in a few years' time will not immediately know what that means.

Thanks for noticing this. We have now edited as suggested: “Although flu circulation and disease were very low during 2020/21 and 2021/22, this was likely driven by COVID-19 control measures [41]. However, most COVID-19 control measures have since been suspended, and winter 2022/23 has seen high levels of SARS-CoV-2 and flu virus co-circulation and increased flu-related hospitalisations.” (L409)

**Reviewer #2:**

General feedback

This paper focuses on the socio-economic inequalities associated with uptake of influenza vaccination in the Greater Manchester Region, UK. The authors conducted their statistical analyses with data spanning several subsequent years of flu seasons, the most recent two taking place during the COVID19 pandemic (2020-21 and 2021-22). They used income deprivation as the primary exposure and study three separate age groups: preschool, primary and older (>=65 years) individuals. Their results show that the least deprived populations have a higher uptake of influenza vaccination compared to the most deprived groups across the years. This gap increased in all age groups after the onset of the pandemic due to an increased uptake in the least deprived group compared to the most deprived group. The authors conducted several sensitivity analyses.

This study makes use of a rich dataset and presents useful findings to advance knowledge on inherent inequalities in access to influenza vaccination. The paper is clearly presented and written, and includes a variety of illustrative figures and detailed tables.

Specific comments to each section of the paper

1.	Abstract

a.	It is not clear what the uptake gap represents. This becomes clear when reading the Results section (absolute difference in % uptake). Can you update the abstract with a clearer definition of ‘uptake gap’?

Thank you for pointing this out. We have updated as follows: “Amongst older adults, the SII (i.e.: the gap in uptake between the least and most income-deprived areas) doubled over the seven seasons from 8.48 (95% CI [7.91,9.04]) percentage points to 16.91 (95% CI [16.46,17.36]), with approximately 80% of this increase occurring during the pandemic.” (L44)

2.	Methods

a.	“We allowed the sample to vary for each vaccination season based on eligibility (age) and deaths (Figure S1).” : This sentence would read better as “The sample varied for each vaccination season (…)”, unless the authors can explain more their choice of wording.

We agree this is clearer and have edited as suggested “The sample varied between vaccination seasons based on eligibility (age) and deaths (S1 Fig).” (L118)

b.	Main statistical analysis: Why was a time to event outcome (time until vaccination) analysis preferred over a binary outcome (vaccination yes/no) analysis?

We have now added our reasoning to the methods: “We used a time-to-event approach, rather than the binary outcome of vaccination received, because delayed vaccination is also a component of the overall vaccine uptake inequality, with earlier vaccination able to offer protection throughout the peak of the winter flu season.” (L171)

c.	What baseline date what used to calculate the time to event outcome? Was it the beginning of the flu season for each year or a specific day of the year?

Thank you for noticing this omission. We have added: “We estimated associations between income deprivation decile and time-to-vaccination (from 1st September each season)” (L165)

3.	Results

a.	The Figures in the Results section are great illustrations to display the findings, especially Figures 1 and 2.

Thank you!

b.	“To compare trends in inequalities over time, we restricted our sample to groups that were consistently eligible for flu vaccination over multiple seasons, focusing on pre-school children, a subset of primary school children over a subset of seasons, and older adults aged 65 plus.” It’s unclear what this sentence means for each age group. For the older age group, does it mean the sample is restricted to individuals who are >=65years old in 2015/16? If so, I don’t know how this would translate for the 2 younger age groups. Or does it mean the sample is restricted to seasons when each age group is eligible for vaccination? In this case the sentence could be reworded: pre-school and >=65 are included for all seasons, and primary school are included for 2018/19 to 2021/22.

We agree this was unclear. Our intended meaning was that we applied consistent eligibility criteria across all seasons, not that individuals need to be consistently eligible. We have edited as follows: “To compare trends in inequalities over time, we applied vaccine eligibility criteria that were consistent over the study period as indicated by the highlighted boxes in Figure 1. We included pre-school children (aged 2-3), and older adults aged 65 plus. Given the staggered vaccination rollout in primary schools, we used a subset of primary school ages (aged 4-9) over a shorter range of seasons (2018/19-2021/22).” (L217)

In combination with the edits to L118 (response to comment above) this should make the inclusion criteria clear.

c.	Tables S1-S13: Should the label say “adjusted by income-deprivation and by age”?

We have updated all figure and table titles as suggested. For example: “S1 Table: Relative age-adjusted income deprivation-related inequalities in flu vaccine uptake amongst pre-school children (age 2-3). Results from Cox proportional hazards models adjusted by age are reported as hazard ratios with 95% confidence intervals. The reference groups are D10 (least deprived areas) and aged 2 years for each season. The vertical line indicates the onset of the pandemic. Results also shown in Figure 2 in the main text.”

d.	Table S5 seem identical to Table S2, except for the number of observations. Is this because there are very few additional observations included in S5?

Yes, this is because very few young children died during the study period. While it is very unlikely results might change given that, we decided to include it for completeness.

e.	Comparing Table S9 to S3: the hazard ratios for D1 are slightly lower in 20/21 and 21/22, so there is a less sharper increases in inequality after the onset of the pandemic. Could this warrant the results been sensitive to the choice of deprivation measure for the older age group?

Thank you for pointing this out. We have updated the results text to reflect this: “…and were generally robust to using total IMD as an alternative deprivation measure (S10 Table, S11 Table, S12 Table), though estimated changes in inequalities were slightly smaller amongst older adults when using total IMD (S12 Table).” (L254)

f.	“However, amongst pre-school children, excluding those who may have been in primary school resulted in estimates of inequality that were relatively consistent across all vaccination seasons (Table S10)”: what are the changes between S1 and S10 related to this comment?

The meaning here was unclear, so we have rephased as follows: “However, amongst pre-school children, excluding those who may have been in primary school resulted in estimates of inequality that were no-longer statistically significantly different across vaccination seasons (S13 Table)” (L258)

g.	“Inclusion of people clinically eligible for flu vaccination and those eligible due to expanded 2021/22 age criteria indicated inequalities were similar compared to the restricted main study population (Table S13).”

Response g&h combined (please see below).

h.	“Inclusion of people (…) eligible due to expanded 2021/22 age criteria indicated inequalities were similar compared to the restricted main study population (Table S13). Is this referring to the comparison of Table S13 0.56 (D1) to Table S3 0.63 (D1) ?

Response g&h combined: Thanks for raising this, the comparison was previously imprecise. We have therefore added 2 new columns to S16 Table (old Table S13) so that there is now a direct comparison across (1) the main sample (2) expanded age eligibility for 2021/22 and (3) expanded age eligibility and clinical eligibility. This revealed that inequalities across the expanded sample were slightly wider than in the main sample. We’ve edited the main text as follows:

Results: “Estimated inequalities in 2021/22 were slightly wider following inclusion of those eligible due to expanded age criteria in 2021/22 (OR most deprived 0.54 (95% CI [0.54,0.55])) and people clinically eligible for flu vaccination (0.56 (95% CI [0.55,0.56])) compared to the restricted main study population (0.57 (95% CI [0.56,0.58])) (S16 Table).” (L260)

Discussion: “Sensitivity analysis including current clinical eligibility and expanded age eligibility for the 2021/22 season suggested our results likely slightly underestimate the full extent of uptake inequalities across the total population.” (L360)

**Reviewer #3:**

Thank you for asking me to review this paper. It is an interesting topic, may help inform future flu vaccination roll-outs and highlights important findings around widening social inequality.

The abstract the clearly written.

Introduction

Overall clearly written

Line 46 - could you expand here to give some potential cited reasons for lower vaccine uptake in more deprived neighbourhoods? Some general reasons here would be useful for context, before moving onto COVID-19 pandemic specific context.

Thanks for this helpful suggestion. We’ve now expanded this section of the introduction to add reasons previously cited in the literature: “Possible explanations for these observed inequalities include differential health knowledge and information, with understanding of the risks of flu infection, perceived vulnerability, and concerns about flu vaccine side effects each potentially differing across socioeconomic groups [5]” (L69)

Could something be mentioned in the introduction/later in the discussion about the effect of COVID-19 related illness (and long-COVID) and potential influence on flu vaccination uptake?

This is a really interesting point. We have added this to the discussion (please see below).

Methods

Line 124: I would like more information on how this data is recorded/collected.

We agree this is helpful as readers may not be familiar with the data sources for these indices. We have now added detail to the methods text and cited the technical report which has further details: “Specifically, these measures are derived from the proportion of children aged 0 to 15 (IDACI) or older adults aged 60 plus (IDAOPI) in each LSOA who are living in income-deprived households, which is defined by receipt of income-assessed benefits or tax credits with an equivalised income of less than 60% of the national median (before housing costs) [25]. These statistics are based on administrative records held by the Department for Work and Pensions and HM Revenue & Customs [25].” (L138)

Results

The results might be better structured with 3 separate subheadings to describe the three very different groups.

We appreciate this suggestion but have decided to keep the current structure (relative then absolute inequalities) for consistency with the methods used and figures. However, we agree the clarity could be improved by structuring so have split paragraphs so that each paragraph now only discusses one age group.

34.9% pre-school uptake seems very low? There are brief reasons described to explain this in the discussion, but this point requires further expansion in the discussion including correlation with previous studies.

Thank you for raising this important point, we have now expanded this section with comparison to the literature: “Our estimates of overall flu vaccine uptake within GM are consistently lower than national uptake estimates [6,33], which is likely due in part to above average income deprivation within the region. Estimated uptake was particularly low amongst pre-school children (34.9% in 2021/22), which was also substantially lower than estimated national uptake (50.1%) [33]. However, our estimates of uptake amongst this age group are consistent with published data from Merseyside, a similarly deprived area in the North West of England, where almost 80% of neighbourhoods had less than 35% flu vaccine uptake amongst pre-school children in 2015/16 [5]. The authors suggest that this may be in part due to lack of parental awareness of the risks of flu infection to young children, despite relatively high rates of hospitalisations amongst this age group [5]. However, low estimated uptake amongst children may also be partly driven by issues with assigning eligibility based on age at the index date discussed in more detail below, and we cannot exclude the possibility of some missing vaccination records in our dataset.” (L330)

Limitations

A suggested limitation in this study is lack of further information on other socio-demographic factors such as ethnicity. Ethnicity and cultural beliefs have been associated with lower vaccine uptake and higher morbidity/mortality from COVID-19 - it would have been informative to see this data and how it ties in with the widening inequalities by social deprivation, particularly in this urban area of GM

We agree that this is a limitation and have added the following to the discussion: “Although we did not find evidence that socioeconomic inequalities in flu vaccine uptake varied by sex across any age group, there are likely other intersecting axes of health inequality that may contribute to overall inequalities in flu vaccine uptake. For example, a limitation of our analysis is that we did not explore inequalities by ethnic group, which is a known determinant of both flu vaccine and COVID-19 vaccine uptake in this study population [24]. Given that structural racism results in people from ethnic minority backgrounds being over-represented in more deprived areas [34], our results raise the possibility that ethnic inequalities in flu vaccine uptake may also have widened, which will be an important area for future research.” (L363)

And “Further research is needed to understand whether inequalities along other sociodemographic axes of inequality and for other routine vaccinations have also widened.” (L419)

Comparison to literature

This section is very brief.

Line 303 - suggest expand here as to what previous reports have said about pre-pandemic inequalities.

As described above, need to expand more on previous study findings in relation to each of the three groups (particularly pre-school)

Responding to these three points together, we have now expanded the comparison to the literature section. This includes the discussion of low uptake amongst pre-school children (L330-341 in response to comment above) as well as a longer discussion of pre-pandemic inequalities and potential explanations for socioeconomic differences:

“The moderate to large pre-pandemic socioeconomic inequalities in flu vaccine uptake are consistent with previous reports [4,5]. Existing research has suggested that these inequalities may be partly explained by differences across socioeconomic groups in health knowledge about the potential consequences of flu infection and about vaccine effectiveness and safety [5]. Previous studies also found that although flu vaccine uptake is usually highly correlated with previous flu vaccine uptake [28], the COVID-19 pandemic was associated with increases in people intending to take up vaccination for the first time [14]. In a large survey, the most frequently reported motivations for first-time flu vaccine uptake were to protect oneself – both from flu directly and from coinfection with SARS-CoV-2 – as well as uptake being viewed as ‘sensible’ and a way to protect healthcare capacity during pandemic pressures [14]. Given that older adults are more vulnerable than children to severe COVID-19 [29], these changes in vaccine uptake intention may apply more to the older adult population, potentially explaining the wider increases in inequalities amongst this group. Conversely, the lower risk of severe COVID-19 amongst children led to substantial debate about the relative risks and benefits of vaccinating children and adolescents against COVID-19 [30,31], with the UK being slower to offer vaccination to under 18s than some other high-income countries [32]. These policy debates may have highlighted the potential risks of vaccination to parents, potentially also increasing parental uncertainty about other vaccines during childhood.” (L316)

Overall, the conclusion considers a broad range of reasons for widening socio-economic inequalities with flu vaccine uptake during this period. There are 2 points I think should be covered that are missing:

-	Those from more deprived backgrounds experienced much higher rates of COVID-19, morbidity and mortality (including long-Covid) - could this have affected uptake of flu vaccine afterwards?

Thank you for this interesting suggestion. We have added to the discussion as follows: “Another factor that could have affected inequalities in access to flu vaccination are the differential impacts of long-term ill-health due to long COVID [36] and pandemic-related delays in elective health care [37]. While increased morbidity may have increased some people’s perceived need for flu vaccination, worsening health might also have increased the difficulty of accessing vaccination. Given the higher burden of both long COVID [36] and long-term health conditions [38] amongst people living in more deprived areas, these factors could have contributed to widening inequalities.” (L383)

-	There is some discussion of misinformation, but not much around "vaccine hesitancy" and mistrust, which is prevalent amongst patients from more deprived backgrounds and minority ethnic groups - which was exacerbated during the COVID-19 pandemic; also vaccine hesitancy amongst parents around the time of the COVID-19 vaccine being introduced.

Suggested reference for further https://journals.plos.org/plosmedicine/article?id=10.1371/journal.pmed.1003826 context: - though there are others more relevant to this group - but this point is worth expanding on.

Thank you for raising this, we agree trust is important for vaccine uptake. We have avoided the use of the term “vaccine hesitancy” as during our public involvement and engagement discussions, this was brought up as a label that some people felt was unhelpful. However, we have now added another reference and discussion about the importance of trust in the choice of which information to accept:

“Similarly, the pandemic was associated with extensive sharing of false conspiracy theories related to the COVID-19 vaccines and the pandemic more broadly, and endorsement of such ideas has been shown to be associated with lower intention to take up vaccination [39]. Trust in public health messaging over misinformation and disinformation is therefore important for vaccine uptake, and there may have been social gradients in exposure to, or responses to, recent pro- and anti-vaccine messaging.” (L392)

We also added some additional discussion about how parental trust in vaccination may have changed during the pandemic: “Conversely, the lower risk of severe COVID-19 amongst children led to substantial debate about the relative risks and benefits of vaccinating children and adolescents against COVID-19 [30,31], with the UK being slower to offer vaccination to under 18s than some other high-income countries [32]. These policy debates may have highlighted the potential risks of vaccination to parents, potentially also increasing parental uncertainty about other vaccines during childhood.” (L326)

Overall this is a well executed study and clearly written paper with some important points on widening socioeconomic inequalities that would be valuable to those involved in future vaccination planning/ outreach to underserved communities.

Thank you for all your helpful comments and suggestions.
