# Supplementary material for: Evaluating socioeconomic inequalities in influenza vaccine uptake during the COVID-19 pandemic: A cohort study in Greater Manchester, England
Source: PLoS Med. 2023 Sep 26;20(9):e1004289. doi: 10.1371/journal.pmed.1004289 (PMC10522043; doi:10.1371/journal.pmed.1004289)
Supplement: Attachment — Submitted filename: responsetoreviewers.docx [file pmed.1004289.s030.docx]

We are very grateful to the editors and reviewers for their additional comments. We have revised the manuscript as indicated below and hope it will now be considered ready for publication.

**Requests from Editors:**

GENERAL

Thank you for considered and detailed responses to editor and reviewer comments.

Please see below for further minor points that we request you respond to in full.

Please revise your manuscript using either British English or American English, e.g., colored/coloured (Description Figure 1) or age-standardised/age-standardized (Line 267).

We have used British English in all cases

Please choose a consistent spelling for the terms “income-deprived”/”income deprived” as well as “income deprivation”/”income-deprivation” and revise you manuscript accordingly.

We have now used income-deprived and income deprivation consistently throughout

Please ensure to use a consistent tense within a section of the manuscript.

We have checked and edited as needed throughout

Please add 'years’ to ages stated e.g. age two to three years, throughout your manuscript

We have added this throughout, including in supplementary figures and legends. E.g.: L39 “(age two to three years), primary school children (age four to nine years), and older adults (age 65 years plus)”

**ACADEMIC EDITOR COMMENTS**

The manuscript has much improved and I am happy with the revised version.

TITLE

Please revise your title according to PLOS Medicine's style. Your title must be nondeclarative. We suggest “Evaluating socioeconomic inequalities in influenza vaccine uptake during the COVID-19 pandemic: A cohort study in Greater Manchester, England” or similar

We have revised the title as suggested

ABSTRACT

Line 33: Please temper assertions of primacy ("no existing studies have investigated…”) by adding ‘to the best of our knowledge’ or similar.

We have added “to the best of our knowledge”

Line 38-9: Please add 'years’ to ages stated e.g., age two to three years.

We have added this throughout

Lines 43-51: Please add the according unit (e.g., percentage points) to numbers occurring in your abstract.

We have added in all cases now. L44: “Amongst older adults, the SII (i.e.: the gap in uptake between the least and most income-deprived areas) doubled over the seven seasons from 8.48 (95% CI [7.91,9.04]) percentage points to 16.91 (95% CI [16.46,17.36]) percentage points, with approximately 80% of this increase occurring during the pandemic. Before the pandemic, income-related uptake gaps were wider amongst children, ranging from 15.59 (95% CI [14.52,16.67]) percentage points to 20.07 (95% CI [18.94,21.20]) percentage points across age groups and vaccination seasons. Amongst pre-school children, the uptake gap increased in 2020/21 to 25.25 (95% CI 24.04,26.45]) percentage points, before decreasing to 20.86 (95% CI [19.65,22.05]) percentage points in 2021/22. Amongst primary school children, inequalities increased in both pandemic years to reach 30.27 (95% CI 29.58,30.95]) percentage points in 2021/22.”

AUTHOR SUMMARY

The Author Summary should immediately follow the Abstract in your revised manuscript.

We have moved the author summary to the correct position

In the third bullet point under ‘Why was this study done?’, please temper assertions of primacy ("no existing studies had analysed…”) by adding ‘to the best of our knowledge’ or similar.

We have added this qualification

Please revise the second bullet point under “What did the authors do and find?”. Editorial suggestion: “We focused on young children and older adults, as these age groups are at higher risk of severe outcomes from flu infection and are eligible for annual flu vaccination provided free at the point of service.”

We have edited as requested

In the final bullet point of ‘What Do These Findings Mean?’, please describe the main limitations of the study in non-technical language.

We have changed the final bullet point to (L88) “A limitation of this study is the use of a local dataset for Greater Manchester, so although this is a relatively diverse region our results may not be generalisable to other areas.”

INTRODUCTION

Lines 65-66 suggest: “[…] (young children and adults who are aged over 65, groups with certain preexisting health conditions, or pregnant people [3]).

We have edited as suggested

Lines 74-76 suggest: “Flu vaccine uptake amongst pre-school and school aged children has consistently been lower than amongst older adults, ranging from approximately 30% to 60% pre-pandemic.”

We have edited as suggested

Line 90: Please write million instead of ‘M’.

We have edited as suggested

Lines 93-94 suggest: “[…], despite overall higher levels of deprivation than the national average [21].”

We have edited as suggested

Lines 94-95 suggest: “For these reasons, Greater Manchester is a useful setting to study changes in socioeconomic inequalities in vaccine uptake.”

We have edited as suggested

METHODS

Line 110: Please change “roll-out” to “rollout” since you have used the latter spelling consistently throughout your manuscript.

Correct to rollout

Line 131: Please define the following abbreviation: UK.

Added (L162) “United Kingdom”

Line 142: Please define the following abbreviation: HM (HM Revenue & Customs)

Added (L173) “His Majesty’s”

Please revise the following statement for improved clarity: “In regression analysis age in years was used for children, then age was grouped into five-year age bands up to 80 plus for adults”. Editorial suggestion: “In regression analysis, age in years was used for children and age grouped into five-year age bands up to 80 years plus was used for adults.”

We have edited as suggested

Please ensure that the study is reported according to the RECORD guideline, and include the completed checklist as Supporting Information. Please add the following statement, or similar, to the Methods: "This study is reported as per the REporting of studies Conducted using Observational Routinely-collected Data (RECORD) guideline (S1 Checklist)." The guideline can be found here: https://urldefense.com/v3/__https://www.record-statement.org/checklist.php__;!!PDiH4ENfjr2_Jw!B47kL4OOE9yQpoN1-GC6jLr2NySFNbcSko3HEEfHI6o7DIbrOauv7gqunmoYyP1m096D5TM8dRwIOmmEDKM_G32gIaA$ [record-statement[.]org] When completing the checklist, please use section and paragraph numbers, rather than page numbers which will likely no longer correspond to the appropriate sections after copy-editing.

We have changed the previous included STROBE checklist for a RECORD checklist and have updated the supplementary file and supporting information caption to reflect this.

We have changed the text in the methods section to reflect this (L233): “We used the REporting of studies Conducted using Observational Routinely-collected Data (RECORD) guidelines to write up this study (S1 RECORD).”

We have also added the following to the methods as required in the RECORD checklist (L184) “All codes, algorithms, and code set validation used to define the populations, outcomes, exposures, and covariates can be found here: <https://github.com/rw251/gm-idcr/tree/master/projects/025%20-%20Watkinson>”

RESULTS

Table 1: We suggest changing the table title to “Table 1 – Baseline study population statistics.”

We have edited as suggested

Line 214 suggest: “Figure 1 […]” instead of “Fig 1[…]”.

We have edited as suggested

Figure 2: Please add a close bracket in the figure legend changing “(Most deprive” to “(Most deprived)”.

We have corrected this

S2 Table/S4 Table/S6 Table: In your table description, you describe the results as adjusted by age (“Results from Cox proportional hazards models adjusted by age are reported as hazard ratios with 95% confidence intervals.”). However, the results presented are unadjusted by age as described in the main manuscript. Please revise accordingly.

We have revised in the table description in each supplementary file and at the end of the main text. The results are unadjusted by age.

Lines 258-258 suggest, “However, amongst pre-school children, excluding those who may have attended primary school resulted in estimates of inequality that were no longer statistically significantly different across vaccination seasons.”

We have edited as suggested

Lines 262-264: Please cite the supplementary tables S17 Table – S22 Table at the end of the sentence.

We have added the citation (new L298)

S21 Table/S22 Table: Please remove “Results also shown in Figure 2 in the main text.” from your table description.

We have removed this

Line 269: Supplementary table S17 appears to be wrongfully cited here. Please check and revise throughout your entire manuscript (S23 should be cited instead).

Thank you. We have corrected the table citations throughout

Figure 3: In Figure 3C, please show the axis beginning at zero. If this is not possible, please show a break in the axis.

Due to the ongoing cyber attack at the University of Manchester, access to the dataset has been revoked for security reasons and we’ve been advised that this is likely to continue for several months. We were therefore unable to edit the graph in Figure 3C. We have manually added a break in the axis to indicate that the axis does not start at zero. We apologise for this inconsistency in Figure 3 and hope that this will be acceptable given the circumstances.

Line 270-272/Line 284: Please add units to all numbers occurring in your manuscript when applicable (instead of ‘19.82’ it should be ‘19.82 percentage points’). Please check throughout your entire manuscript.

We have added units throughout

Line 276/281/285/287: Supplementary tables S17 and/or S18 appear to be wrongfully cited here. Please check and revise throughout your entire manuscript (S23 and/or S24 should be cited instead).

Thank you. We have corrected the table citations throughout

DISCUSSION

Please remove the subheadings from your Discussion.

We have removed the subheadings

Lines 328-328 suggest: “Conversely, the lower risk of severe COVID-19 in children has led to considerable debate about the relative risks and benefits of vaccinating children and adolescents against COVID-19 [30,31], with the UK being slower than other high-income countries to offer vaccination to those under 18 years [32].”

We have edited as suggested

Lines 330-331 suggest: “Our estimates of overall flu vaccine uptake within GM are consistently lower than national uptake estimates [6,33], likely in part due to above-average income deprivation within the region.”

We have edited as suggested

Lines 344-346 suggest: “A strength of this study is the use of a dataset with almost population-level coverage for a large city region [24], along with robust measures of income deprivation that specifically capture the proportion of children or older adults living in poverty at a neighbourhood level [21].”

We have edited as suggested

Lines 353-354 suggest: “Sensitivity analysis indicated that this is unlikely to have affected the results for primary school children or older adults.”

We have edited as suggested

Lines 358-359 suggest: “While this could introduce bias, sensitivity analysis excluding those who died at any point in the study period indicated that this is unlikely to have substantially affected estimates of inequality.”

We have edited as suggested

Line 367: Please change “over-represented” to “overrepresented”.

We have corrected this

Line 383-384 suggest: “Another factor that may have affected inequalities in access to flu vaccination is the differential impact of long-term ill health due to long COVID [36] and pandemic-related delays in elective health care [37].”

We have edited as suggested

REFERENCES

Please ensure that journal name abbreviations match those found in the National Center for Biotechnology Information (NCBI) databases (https://urldefense.com/v3/__http://www.ncbi.nlm.nih.gov/nlmcatalog/journals__;!!PDiH4ENfjr2_Jw!B47kL4OOE9yQpoN1-GC6jLr2NySFNbcSko3HEEfHI6o7DIbrOauv7gqunmoYyP1m096D5TM8dRwIOmmEDKM_J-CAos4$ [ncbi[.]nlm[.]nih[.]gov]), and are appropriately formatted and capitalised.

We have checked all abbreviations and formatting are correct

Where website addresses are cited, please specify the date of access.

We have checked all website links and updated today as the date last accessed

SOCIAL MEDIA

To help us extend the reach of your research, please provide any Twitter handle(s) that would be appropriate to tag, including your own, your co-authors’, your institution, funder, or lab. Please detail any handles you wish to be included when we tweet this paper, in the manuscript submission form when you re-submit the manuscript.

Twitter handles: @RuthWatkinson @HOPE_UoM @ARC_GM_ @MattXSutton @dukester24 @Mons93Stephanie

**Comments from Reviewers:**

**Reviewer #1: Alex McConnachie, Statistical Review**

I thank the authors for their consideration of my original points, and I am happy with their responses, I have no further comments to make.

Many thanks for your very helpful original points. We are glad our responses addressed your comments.

**Reviewer #2: General comment**

Thank you for inviting me to review this article.

The revised draft of the paper reads very well. The authors have addressed in detail the previous set of reviews.

We are grateful for your thoughtful and careful review of both our original and revised manuscripts. We have now addressed the remaining comments below.

Specific comments

L159 - The acronym SII is not defined.

We have now added L193: “slope index of inequality (SII)”

L176 - This sentence of the main analysis mentions 'excluding those who died before or during each season' while L180-181 says 'We then restricted the main analysis sample to those who remained alive throughout each vaccination season.', which is part of the sensitivity analysis. What is the difference between these two analyses?

Thank you for highlighting this. In the first instance the exclusion refers to handling of deaths in the absolute inequalities analysis (age-standardised uptake and slope index of inequality). In the second instance (sensitivity analysis) this is a sensitivity check of the impact of excluding those who died at any point across all vaccination seasons on results from the Cox regression where death is included as a censoring event in the main analysis. We have added the following clarification to the sensitivity analysis section (L215): “We first repeated the main analysis (Cox regression) without adjustment by age. We then restricted the main analysis sample to those who remained alive throughout all seven vaccination seasons.”

Line 203-210: The numerical data reported do not exactly match those in Table 1. Please could you check them again. For example, 70426 children aged 2-3 years are reported while Table 1 indicates a total population of 70419; another example, 16613 children aged 2-3 years in most deprived group vs 16612 in the table.

Thank you for noticing these errors. We have corrected the text as follows (L238-245): “Amongst those eligible for flu vaccination based on age during the 2021/22 vaccination season, there were 70,419 children aged two to three years (referred to as pre-school children); 233,277 children aged four to nine years (referred to as primary school children); and 454,787 adults aged 65 years plus (referred to as older adults) (Table 1). Income deprivation was higher in GM than the England average, with approximately 24% of pre-school (16,612) and primary school (55,478) children living in the 10% most deprived neighbourhoods nationally. Older adults in GM also disproportionately lived in income-deprived areas (66,568, 14.6%) (Table 1). Flu vaccine uptake was higher in older age groups, with 72.8% of older adults (331,092), 51.1% of primary school children (119,223), and 34.9% of pre-school children (24,575) receiving flu vaccination.

Line 264: Tables S17-22 are not cited.

We have added this

Line 267-87: Tables 23 & 24 should be cited instead of tables 17 & 18.

Thank you, we have corrected this error and checked references to tables throughout
