# Supplementary material for: Evaluating socioeconomic inequalities in influenza vaccine uptake during the COVID-19 pandemic: A cohort study in Greater Manchester, England
Source: PLoS Med. 2023 Sep 26;20(9):e1004289. doi: 10.1371/journal.pmed.1004289 (PMC10522043; doi:10.1371/journal.pmed.1004289)
Supplement: Attachment — Submitted filename: r2r.docx [file pmed.1004289.s031.docx]

We have now responded to all comments from the editors as outlined in the point-by-point responses below.

Please check your Competing Interest statement. In the Financial Disclosure statement, you have stated that one of the authors, Matt Sutton, is a Senior Investigator at the NIHR. As stated on our website, a “Relationship (paid or unpaid) with organizations and funding bodies including nongovernmental organizations, research institutions, or charities” is considered a non-financial competing interest.

All authors must declare their relevant competing interests per the PLOS policy, which can be seen here:

https://urldefense.com/v3/__https://journals.plos.org/plosmedicine/s/competing-interests__;!!PDiH4ENfjr2_Jw!AWUI2H2HBvm8tCviH70cbLPoYObi8wpmoYKtsZBfLknzrnFTRa3ln07gL725M4mIMrUEHbjgQUIhmxse2XtUKo1_O10$ [journals[.]plos[.]org] For authors with ties to industry, please indicate whether any of the interests has a financial stake in the results of the current study.

We have now expanded both the financial disclosure and competing interests statements to include the following information about Matt Sutton’s role as a Senior Investigator at the NIHR:

“MS is an NIHR Senior Investigator, whose collective role is to: contribute significantly to NIHR as a senior leader; lead in training and development of NIHR’s people; act as an ambassador for the NIHR in the wider system; demonstrate research excellence; contribute to growth; integrate patient, care users, carers and public involvement and engagement in research; and play a leading role in NIHR’s contribution to growth. Each Senior Investigator receives a discretionary award of £20,000 per year of appointment to fund activities that support their research. Within the last five years, MS has had the following roles for NIHR: National Lead for Economics in the NIHR Applied Research Collaborations, Chair of the Economics Workstream for the NIHR Methodology Incubator, Chair of the NIHR Economics Group; Member of the NICE Implementation Strategy Group; Member of the Rapid Service Evaluation Team 2023 Committee; Member of the School for Social Care Phase IV Committee; Deputy Chair of the NIHR Advanced Fellowships Panel; Deputy Chair of the NIHR Health Services and Delivery Research Funding Committee; Member of the NIHR Clinician Scientist Fellowship Panel; Member of the Department of Health Policy Research Programme Board; and Member of five Study Steering Committees.

The views expressed are those of the authors and not necessarily those of the NIHR or the Department of Health and Social Care. The NIHR had no role in the collection, analysis, and interpretation of data; writing of the paper; and/or decision to submit for publication.”

Please check your manuscript carefully for grammar, spelling and punctuation. Please consider proofreading by a native English speaker.

We have checked carefully and made some additional edits (using tracked changes).

Title: Please revise your title to replace 'flu' with 'influenza'. We suggest “Evaluating socioeconomic inequalities in influenza vaccine uptake during the COVID-19 pandemic: A cohort study in Greater Manchester, England”.

We have updated the title as requested.

l.54: In the last sentence of the Abstract Methods and Findings section, please describe the main limitation(s) of the study's methodology or change “A limitation” to “The main limitation”.

We have edited as suggested.

l.95: Please define ‘NHS’.

We have added “National Health Service (NHS)”.

ll.123-126 suggest: “Greater Manchester is also a socioeconomically diverse area, including some of the least deprived neighbourhoods nationally, despite overall higher deprivation levels than the national average [21].”

We have edited as suggested.

l.204: Please introduce the abbreviation “CI” for confidence intervals at first use.

We have added “95% confidence intervals (CI)”.

l.307: Please, for add square brackets open for “increased from 15.59 percentage points (95% CI 14.52,16.67]) in 2015/16 to 19.82 (95% CI 18.66,20.98]) percentage”.

l.317: Please, add a square bracket open for “29.58,30.95])”. Please revise throughout your entire manuscript.

Thank you, we have corrected this throughout.

ll.443-444: Please provide a reference.

We have now cited “Surveillance of influenza and other seasonal respiratory viruses in the UK, winter 2022 to 2023” by UKHSA from the gov.uk website (new ref 42).

ll.477-478: Please remove the ‘Declaration of Interest’ statement. These details should only be provided in the according section of the online submission form.

We have removed this.

Please check all figures and tables thoroughly.

We have checked all the figures and tables.

Table 1: Define ‘GM’. The footnotes below the table do not correspond with the footnotes/asterisks in the table. Please revise and check throughout your entire manuscript.

We have replaced GM with “Greater Manchester”.

Thank you, we have now corrected this so that the footnotes correspond. It now reads: “^1^ IQR: interquartile range. ^2^ IDACI (income deprivation affecting children index) used as income deprivation measure for age 2-3 years and age 4-9 years; IDAOPI (income deprivation affecting older people index) used as income deprivation measure for age 65 plus years.”

Figure 1: Please add a unit for ‘uptake’ (%).

We have now added this.

Figure 4/S24 Table: You describe the age-adjusted estimated difference in vaccine uptake between the least and most income-deprived areas as presented in percentage. However, the slope index of inequality is presented in percentage points. Please change the figure/table description and exchange “%” with “percentage points”.

Thank you, we have now corrected this.

Figure S2: Due to the size of the individual graphs, the visibility of the data presented in Figure S2 is poor. Please use a format in which the individual graphs are displayed larger.

We have split this figure over two pages to improve the visibility of the data.

S4 Table: Please remove “[…] and age 4 years for each season.” from the figure description as the results in this table are unadjusted for age (“The reference group is D10 (least deprived areas).”).

Thank you, we have corrected this.

Table S1/S2/S7/S13/S17/S18: Please make sure to add an asterisk following IDACI in the tables.

We have added this.

S16 Table: A third reference group seems to be ‘people not clinically eligible for flu vaccination’. Please add in your table description.

We have now added this and corrected the supporting information caption.
